# Photocontrolled DNA minor groove interactions of imidazole/pyrrole polyamides

**DOI:** 10.3762/bjoc.16.8

**Published:** 2020-01-09

**Authors:** Sabrina Müller, Jannik Paulus, Jochen Mattay, Heiko Ihmels, Veronica I Dodero, Norbert Sewald

**Affiliations:** 1 Organic and Bioorganic Chemistry, Department of Chemistry, Bielefeld University, PO Box 100131, D-33501 Bielefeld, Germany; 2 Organic Chemistry I, Department of Chemistry, Bielefeld University, PO Box 100131, D-33501 Bielefeld, Germany; 3 Organic Chemistry II, Department Chemistry – Biology, Siegen University, Adolf-Reichwein-Str. 2, D-57068 Siegen, Germany

**Keywords:** azobenzene, DNA minor groove, *N-*methylimidazole, *N-*methylpyrrole, photoswitches, polyamide

## Abstract

Azobenzenes are photoswitchable molecules capable of generating significant structural changes upon *E-*to-*Z* photoisomerization in peptides or small molecules, thereby controlling geometry and functionality. *E-*to-*Z* photoisomerization usually is achieved upon irradiation at 350 nm (π–π* transition), while the *Z-*to-*E* isomerization proceeds photochemically upon irradiation at >400 nm (n–π* transition) or thermally. Photoswitchable compounds have frequently been employed as modules, e.g., to control protein–DNA interactions. However, their use in conjunction with minor groove-binding imidazole/pyrrole (Im/Py) polyamides is yet unprecedented. Dervan-type Im/Py polyamides were equipped with an azobenzene unit, i.e., 3-(3-(aminomethyl)phenyl)azophenylacetic acid, as the linker between two Im/Py polyamide strands. Only the (*Z*)-azobenzene-containing polyamides bound to the minor groove of double-stranded DNA hairpins. Photoisomerization was exemplarily evaluated by ^1^H NMR experiments, while minor groove binding of the (*Z*)*-*azobenzene derivatives was proven by CD titration experiments. The resulting induced circular dichroism (ICD) bands of the bound ligands, together with the photometric determination of the dsDNA melting temperature, revealed a significant stabilization of the DNA upon association with the ligand. The (*Z*)-azobenzene acted as a building block inducing a reverse turn, which favored hydrogen bonds between the pyrrole/imidazole amide and the DNA bases. In contrast, the *E-*configured polyamides did not induce any ICD characteristic for minor groove binding. The incorporation of the photoswitchable azobenzene unit is a promising strategy to obtain photoswitchable Im/Py hairpin polyamides capable of interacting with the dsDNA minor groove only in the *Z-*configuration.

## Introduction

The development of small chemical agents to modulate gene expression in an organism has attracted considerable interest in molecular medicine [1]. However, the missing selectivity of chemical agents, such as chemotherapeutics, often results in undesirable cytotoxicity, harming healthy cells, and thereby inducing a multitude of side effects. A major breakthrough concerning selective small DNA-targeting molecules was the use of pyrrole/imidazole hairpin polyamides. They are conceptually derived from the natural products netropsin and distamycin A, which selectively bind to particular sequences of the DNA duplex [2–4]. Covalent tethering of two antiparallel polyamide segments increases the sequence specificity and the affinity of the polyamides to their cognate dsDNA. Different linker strategies were used, with γ-aminobutyric acid (γ) being the most successful representative. The resulting hairpin polyamides bound with 100-fold higher affinity than the corresponding untethered moieties, with the reverse turn module showing selectivity for AT base pairs [5]. The reason for this selectivity was the steric repulsion with the exocyclic amino function of guanine that pointed inside the DNA groove [2]. The group of Dervan has systematically increased the number of binding motifs, and thus achieved sequence-specific binding [6–9]. Selectively binding polyamides adopt an antiparallel hairpin structure where a base pair of the DNA is addressed by a pair of the heterocyclic amino acids in the hairpin assembly. Overall, specific binders have been developed for all possible base pairings (AT, TA, GC, CG; 'pairing rules'). A GC pair is specifically being addressed in the minor groove by *N-*methylimidazole/*N-*methylpyrrole (Im/Py), while AT associates with the pair *N-*methylpyrrole/*N-*methylhydroxypyrrole (Py/Hp). The same applies to CG (Py/Im) and TA (Hp/Py). However, *N-*methylhydroxypyrrole (Hp) is neither easy to synthesize nor sufficiently stable. In practice, therefore, Py/Py is used to address both AT and TA.

Along this line, it is attractive to incorporate a molecular switch for the selective activation of an initially inactive substance, once located at the DNA target, with an external stimulus. Azobenzenes are photoswitchable molecules capable of generating significant structural changes in peptides or small molecules, thereby controlling their geometry and functionality upon irradiation [10–13]. While isomerization of the *E*- to the *Z*-form usually takes place at 350 nm irradiation (π–π* transition), the reverse is photochemically induced at 450 nm (n–π* transition) or thermally. The rate of thermal relaxation, inter alia, depends on the substituents, and only derivatives with very slow thermal relaxation are suitable as bi-stable switches.

Azobenzene and other small photoreactive molecules have been employed as ligands to control DNA or RNA assembly by light [14–20]. Mascareñas et al. reported the first photoisomerizable transcription factor (Tf) that recognized its target sequences by major groove recognition [21–22]. Woolley et al. reported other photoisomerizable Tf mimetics that interacted through the major groove [23–24]. Hybridization of DNA [25–27] or PNA/DNA [28] can be light-controlled by azobenzene-modified DNA. While DNA covalently modified with azobenzene moieties was proven amenable for the light-induced triggering of transcription [24], photoswitchable polyamides that can act as selective DNA minor groove binders displaying tunable affinity have not yet been reported [29–30].

We envisaged that control of the geometry and functionality could be achieved upon replacement of the γ-aminobutyric acid linker between the two hairpin strands by a photoswitchable azobenzene motif. As the azobenzene moiety undergoes a considerable steric reorganization upon isomerization from the *E*- to the *Z*-configuration, the end-to-end distance in a 4,4'-disubstituted azobenzene changes from 9 Å to 5.5 Å upon *E*-to-*Z* isomerization [31–32]. If we consider the minor groove of double-stranded B-DNA to have a width of approximately 5.7 Å [21], the *Z*-isomer of a 4,4’-azobenzene-tethered Im/Py polyamide would fit into the minor groove, while the two polyamide strands establish direct contacts to the bottom of the cognate minor groove. However, previous studies on distamycin A-like derivatives containing only pyrrole moieties and 4,4'-substituted azobenzene as a photoswitchable linker showed that the binding properties of the *Z*- and *E*-isomers were quite similar. Hence, there was no switch in binding activity [29–30]. Importantly, it was demonstrated that the DNA binding was highly dependent on the linker length between the azobenzene and the distamycin moiety.

## Results and Discussion

Here, we report on the design, synthesis, and characterization of photoswitchable minor groove binders based on pyrrole/imidazole hairpin polyamides functionalized with an azobenzene unit in which the *Z*-isomer is sufficiently stable to allow for accurate measurements. Im/Py polyamides bind to the minor groove of DNA in a sequence-specific manner, encoded by antiparallel side-by-side pairs of pyrrole and imidazole carboxamides with higher binding affinities than the pyrrole analogs alone [33–34]. We selected 3-(3-(aminomethyl)phenyl)azophenylacetic acid as the linker between both polyamide strands because it was shown to induce hairpins in peptides upon *E*-to-*Z* photoisomerization [35–36]. According to molecular dynamics calculations, the 3,3'-substituted azobenzenes are more suitable as photoswitchable building blocks to induce a hairpin motif than the 4,4'-substituted correlates [35]. For 3,3'-substituted azobenzenes, the *Z-*form is expected to display higher thermal stability than for the 4,4'-substituted correlates, as the substituents are not conjugated to the N=N double bond [35,37–39]. Moreover, it was demonstrated that in the case of peptides, only the *Z-*isomer adopts the hairpin conformation. In our setup, this could influence the hydrogen bonding contacts between the pyrrole/imidazole amide with DNA bases, helping to achieve proper shape complementarity only when the system is in the *Z-*configuration. Therefore, this azobenzene represents a promising approach to replace the γ-aminobutyric acid linker in the Im/Py polyamide systems.

### Design and synthesis of photoswitchable polyamides

**Scheme 1:**
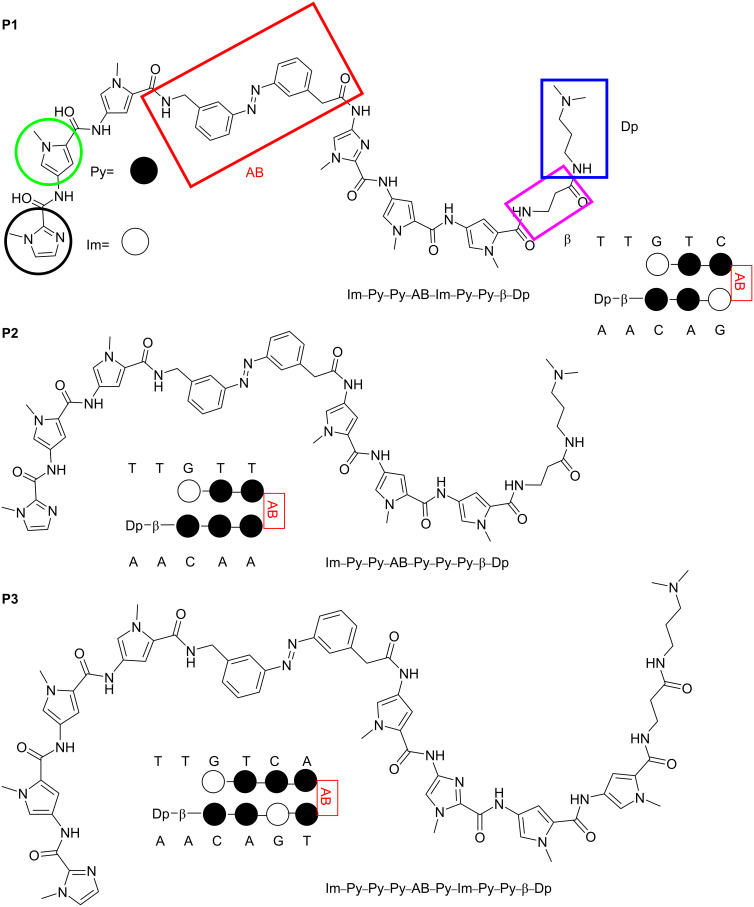
Pyrrole–imidazole–azobenzene polyamides and the dsDNA target sequences employed in this study.

From a pool of new azobenzene-containing polyamides, we present the most promising photoswitchable representatives containing six or eight Im/Py units (**P1**, **P2**, and **P3**, Scheme 1). Their binding capability to dsDNA was analyzed by titration and temperature-dependent circular dichroism experiments.

**Scheme 2:**
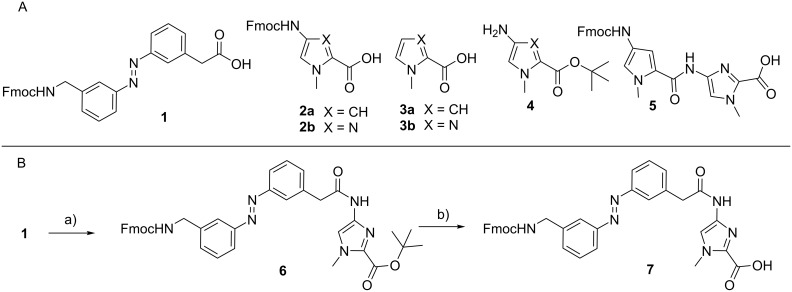
Building blocks required for the synthesis of the photoswitchable Im/Py polyamides. A) Fmoc–Azo–OH **1**, *N-*methylpyrrole and *N-*methylimidazole monomers **2**–**4**, and Fmoc–Py–Im–OH **5**. B) Synthesis of the dimer Fmoc–Azo–Im–OH **7**: a) DCC, THF, rt, 5 min, then **4**, rt, 15 h, 41%; b) BF_3_∙Et_2_O, dichloromethane, 0 °C→rt, 12 h, 73%.

The assembly of the photoswitchable pyrrole/imidazole polyamides (**P1**–**P3**) was performed stepwise on solid phase using the acid-labile 2-chlorotrityl resin and Fmoc as a temporary protecting group, according to the synthetic route published by the Dervan group [40]. The synthesis of the Fmoc-protected photoswitchable ω-amino acid **1** (Scheme 2A) was performed based on a procedure by Aemissegger et al. [35]. The ω-amino acid **1** was subsequently used in the solid-phase synthesis or for the synthesis of the dimer **7** in solution (Scheme 2B).

The incorporation of β-alanine as a 'molecular spring' was required because this allowed for an alignment between hydrogen-bonding groups in long polyamides and in the minor groove of DNA [41].

The Fmoc-protected heterocyclic amino acids **2** were obtained from *N-*methylpyrrole and *N-*methylimidazole, respectively (Scheme 2A). The *N-*terminal *N-*methylpyrrole and *N-*methylimidazole units were introduced by employing diamino derivatives **3** (Scheme 2A) [40]. Moreover, it turned out that coupling the subsequent building block to an *N-*terminal Im building block was problematic because the amino group of the imidazole derivative **2b** is a weak nucleophile, and therefore the Fmoc–Py–Im–OH dimer **5** was obtained [42–43]. Owing to the poor coupling results of Fmoc–Py–OH to the amino function of a terminal Im moiety, the Fmoc–Azo–Im–OH dimer **7** was synthesized in 73% yield by coupling to the amine **4**, as shown in Scheme 2B.

HBTU as a coupling reagent and DIPEA as a base were employed in DMF for the synthesis of **P2**. However, in the case of **P1** and **P3**, the formation of tetramethylguanidinium side products was detected by MALDI–TOF MS. This irreversible *N-*guanylation of the polyamide *N-*terminus resulted from slow carboxy activation of the building block by HBTU and the presence of an excess of HBTU and could not be completely prevented [44–45]. Since this side reaction did not occur with phosphonium salts, **P1** and **P3** were successfully obtained by using PyBOP as an activating reagent [45–46]. After final Fmoc cleavage, the polyamides were released from the resin with a solution of 2.5% TFA in dichloromethane. The free *C-*terminus of the polyamides was modified in solution with *N*,*N*-dimethylaminopropylamine by using PyBOP as an activating reagent to install the corresponding end group. The completeness of all reaction stages was checked by MALDI–TOF MS. Purification of the final products by preparative reversed-phase HPLC gave the TFA salts of the polyamides **P1**, **P2**, and **P3** in 10%, 2%, and 6% yield, respectively (see Experimental section).

### *E*-to-*Z* photoconversion

**Figure 1:**
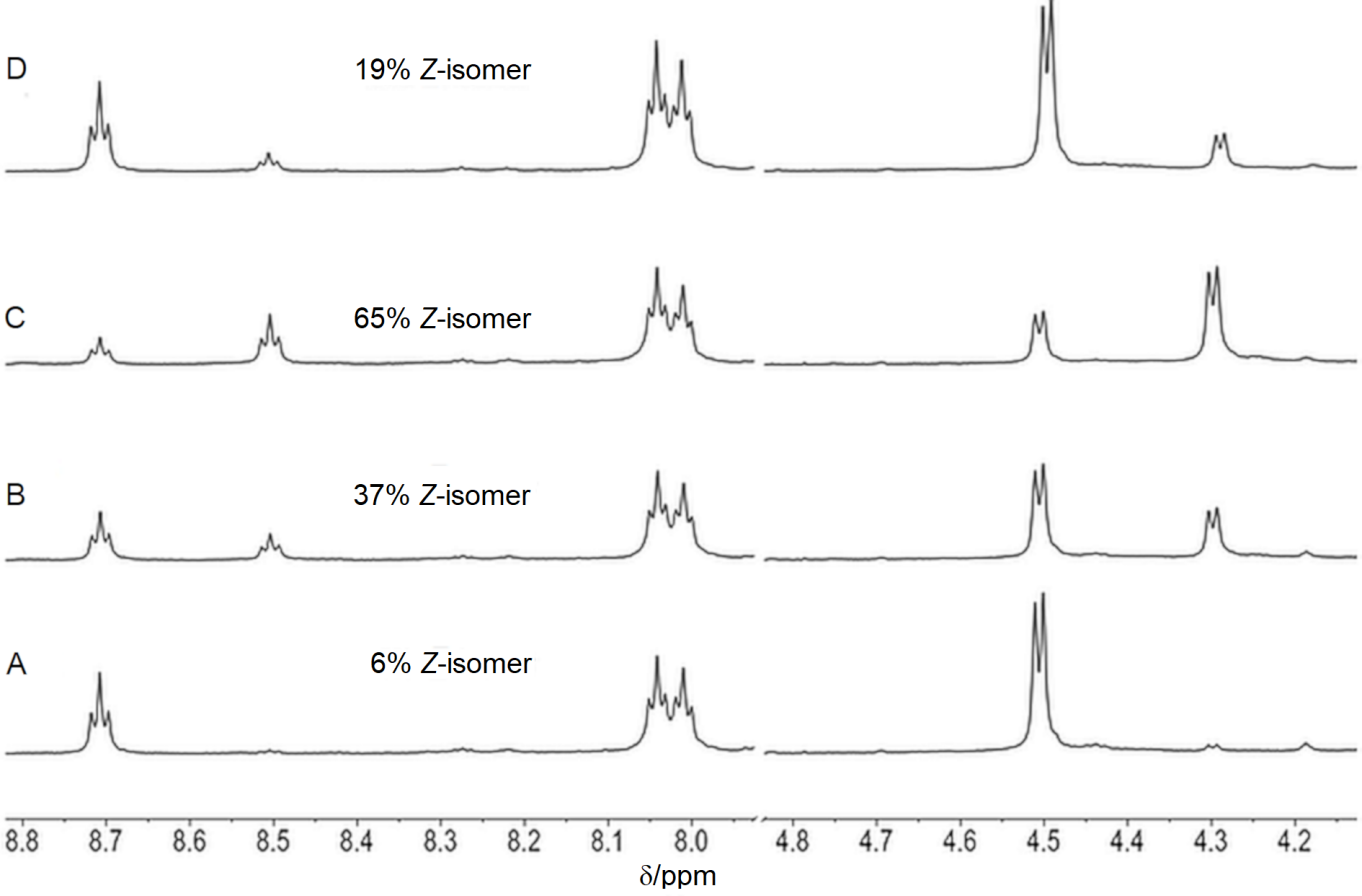
Section of the ^1^H NMR (600 MHz) spectrum of polyamide **P1**. A) Initial thermal equilibrium. B) After irradiation at 350 nm for 10 min. C) After irradiation at 350 nm for 50 min. D) After irradiation at 420 nm for 30 min (in DMSO-*d*_6_, *c* = 8 mM).

The functional properties and photoconversion of 3-(3-(aminomethyl)phenyl)azophenylacetic acid were already established previously [35–36,39]. NMR spectroscopy was employed to quantify the photoconversion of the azobenzene-containing polyamides **P1**–**P3** as well as the stability of the *Z-*isomer. For this purpose, two proton signals were selected, which were not superimposed by other signals and where the chemical shift changed as a result of the switching process. The amide proton of the azobenzene moiety at 8.7 ppm and the adjacent methylene group at 4.5 ppm met these requirements, and hence were used for analysis (Figure 1). The proportions of the individual species in the mixture were determined by integration of the corresponding signals. A representative series of spectra is presented in Figure 1 for polyamide **P1**. In the thermal equilibrium, approximately 6% of the *Z-*isomer was present (Figure 1A).

Upon irradiation at 350 nm, this fraction steadily increased and reached a level of 65% after 50 min irradiation (Figure 1B and Figure 1C). However, the *Z*-isomer content could not be increased further by UV irradiation. The *Z-*isomer could subsequently be switched back to the *E-*isomer (19% *Z*-configuration after 30 min) by irradiation at 420 nm (Figure 1D). The thermal conversion to the *E-*isomer in the dark was slow at room temperature, and even after 18 days, 13% of the *Z-*isomer were still present. The thermal equilibrium (5% *Z*-configuration) was reached only after 40 days in the dark (data not shown).

**Figure 2:**
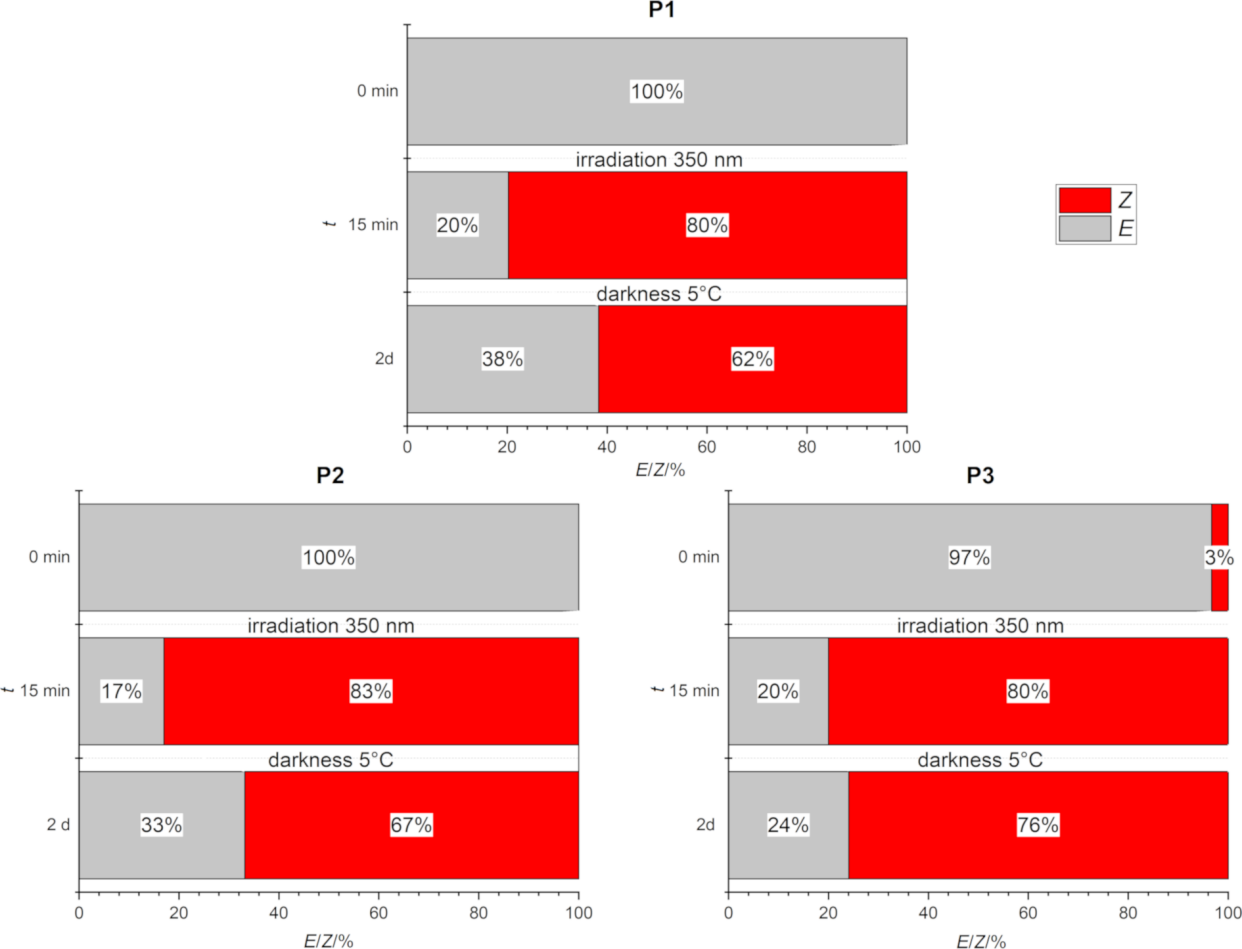
*E*/*Z* isomer ratio of the polyamides **P1**–**P3**. Values were obtained from the respective ^1^H NMR experiments (*c* = 0.80 mM) after irradiation (λ = 350 nm, *t* = 15 min) followed by evaluation after one or two days at 5 °C in the dark. For details, see Supporting Information File 1.

In a previous study, the same azobenzene species incorporated in a hairpin peptide reached a photostationary state of 85% *Z*-isomer [35]. The photostationary state depends on the nature of the azobenzene, but also on the concentration, as the azobenzene is known to form supramolecular assemblies at high concentration, compromising photoconversion [11–12]. When the concentration of polyamide **P1** was reduced to 0.8 mM, 80% of the *Z-*isomer were obtained after 15 min of irradiation (Figure 2). In the case of **P2** and **P3**, similar results were obtained, and the photostationary *Z-*state (83% and 80%, respectively) was reached after 15 min irradiation at 350 nm. It has been reported that the *E*-to-*Z* isomerization of 3,3’-substituted azobenzene compounds proceeds substantially slower than for 4,4’-substituted azobenzene analogs [36]. Moreover, the solutions were kept in the dark to evaluate the thermal stability of the *Z-*isomer, and it was noted that after one or two days, the percentage of *Z-*isomer was slightly reduced (after 2 d: 62% **P1**; 67% **P2**; after 1 d: 76% **P3**).

### Analysis of polyamide–DNA interaction by induced circular dichroism

**Figure 3:**
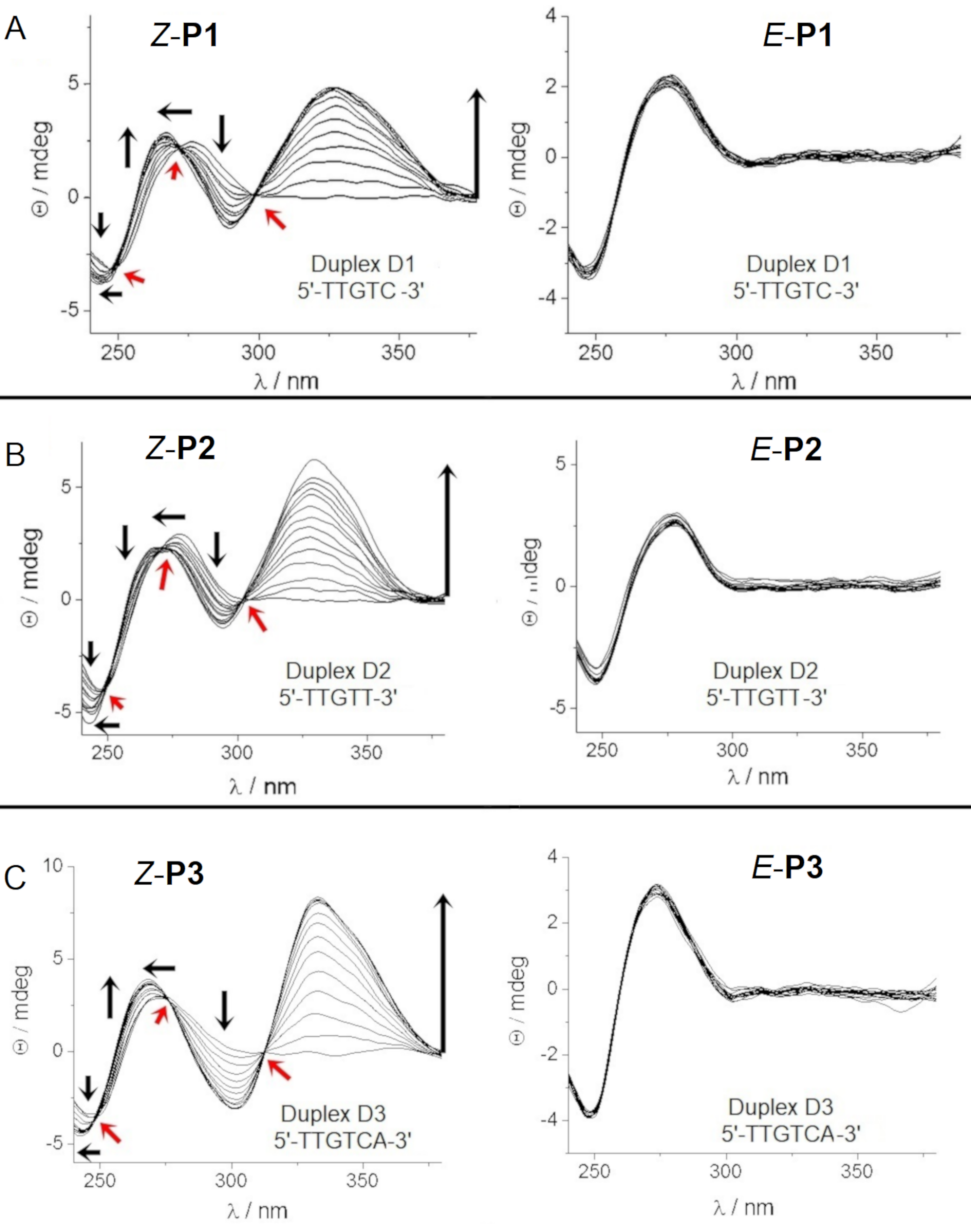
Titration experiments of target DNA sequences with **P1–P3** in the photostationary *Z-*state and the thermal *E*-state. See Table 1 for the complete sequences.

CD spectroscopic analysis is suitable for the characterization of DNA minor groove binders, providing semiquantitative information about binding affinity and thermal stability of the target dsDNA [47–49]. The B-DNA has a positive CD band at 260–280 nm and a negative band at about 245 nm [47]. In contrast, achiral ligands, such as the polyamides, do not give a CD signal. However, by associating the achiral ligand with chiral DNA, an induced circular dichroism (ICD) signal can be detected at wavelengths longer than 300 nm where dsDNA does not absorb. Therefore, the occurrence of ICD is a strong indication of the interaction between ligand and DNA [50–51]. This ICD signal originates from the coupling of the transition dipole moments of the nucleobases and the ligand and is usually positive for polyamide groove binders on B-DNA at about 335 nm [47,52]. Initially, it was difficult to predict, which sequences would be optimal for the new polyamides containing the azobenzene linker. Hence, we employed the target sequences for the Im/Py hairpin polyamides based on Dervan’s pairing rules, such as 5´-TTGTC*-*3´ for **P1**, 5´-TTGTT-3´ for **P2**, and 5´-TTGTCA-3´ for **P3** (Scheme 1). Furthermore, as proof of concept, short hairpin oligonucleotide sequences were employed to investigate the interaction of the polyamides with dsDNA because six- and eight-membered polyamides can only bind in a 1:1 mode, and thus simplify the analysis [41–42,47]. Figure 3 demonstrates that only (*Z*)-polyamides show the characteristic positive ICD signal with the dsDNA sequences tested. The position of the maximum of the positive ICD signal increased with the length of the polyamide (*Z*-**P1** and *Z-***P2**: λ_max_ = 325 nm; *Z-***P3**: λ_max_ = 333 nm), and a new negative band at 280–310 nm was observed, too. The intensity of these new signals increased with increasing concentration of the polyamides. Titrations were performed at molar ratios PA:hairpin DNA from 0 to 4.8, where saturation was reached for *Z-***P1** and *Z-***P3** at the ratio 1:3.3. Considering that only the 1:1 PA:dsDNA binding mode was possible for our setup, the observed behavior was indicative of dynamic binding at the concentration tested. Furthermore, for these compounds, hyperchromicity (increase in signal intensity) and hypsochromic shifts of the B-DNA maximum at ≈275 nm and minimum at ≈250 nm were detected upon addition of polyamide.

Considering that PAs do absorb light at wavelengths < 300 nm, the observed changes cannot be unambiguously attributed to the DNA because the ICD of the PA is combined with the intrinsic CD spectrum of the oligonucleotide. Nevertheless, the hyperchromicity of the signal on increasing PA concentration and the spectral behavior observed were characteristic for a B-DNA structure [51,53].

**Figure 4:**
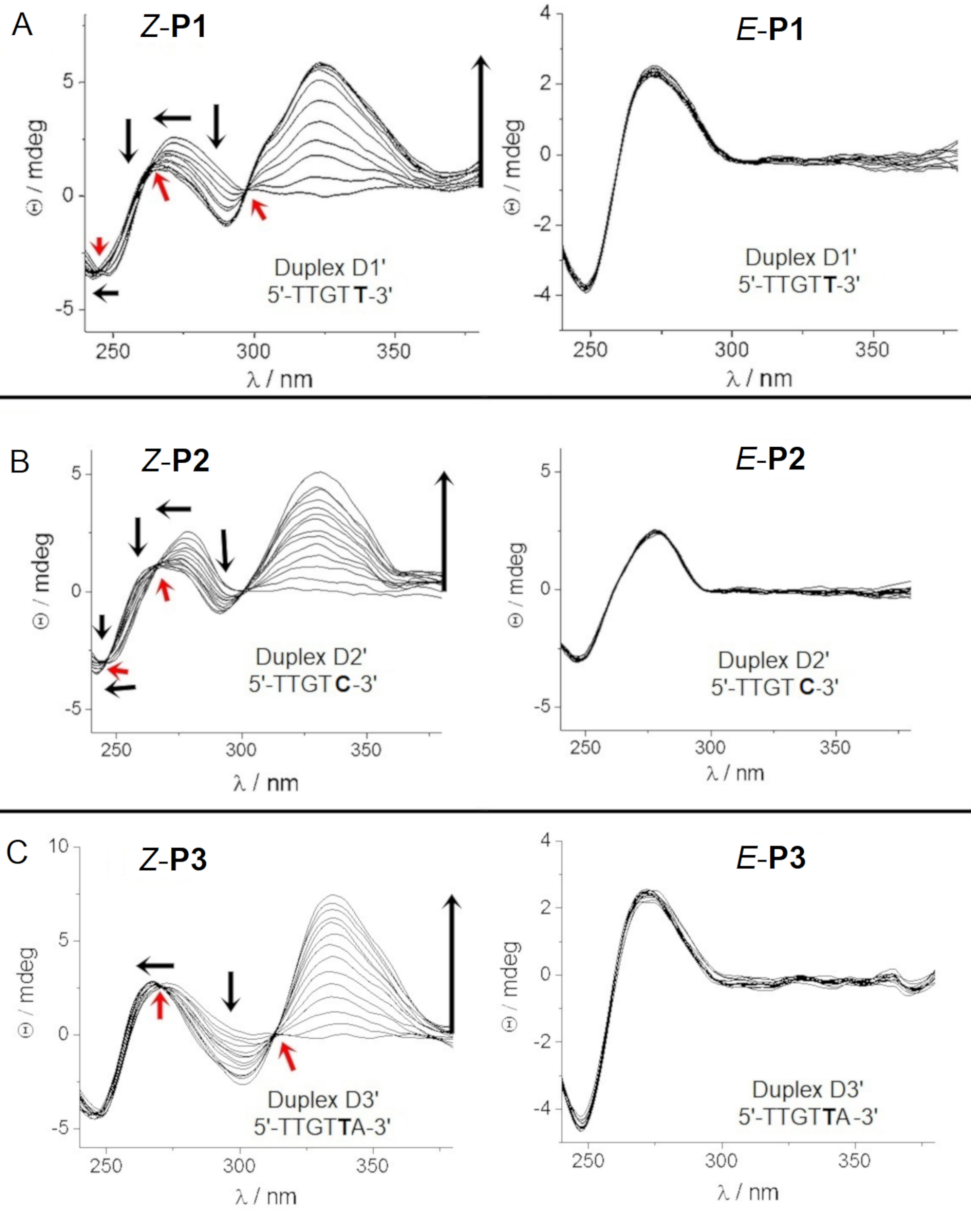
Titration of DNA containing single mutations (in bold) with **P1**–**P3** in the photostationary *Z-*state and the thermal *E-*state. See Table 1 for complete sequences.

In contrast, *Z-***P2** did not reach saturation in the tested concentration range (Figure 3B), which is indicative of a lower binding affinity in comparison with the other PAs. For *Z-***P2**, hypsochromic shifts of the B-DNA maximum at ≈275 nm and the minimum at ≈250 nm upon addition of polyamide were accompanied by slightly reduced hyperchromicity. The appearance of clear-cut isodichroic points in the CD titration (red arrows in Figure 3) suggests the formation of one dominant type of DNA/PA complex, which, together with the positive ICD bands observed at wavelengths > 300 nm, indicate minor groove binding [51,53]. Similar results have been obtained by Wang et al., who evaluated the interaction between hairpin Im/Py PA and short hairpin dsDNA sequences [51–53]. Strikingly, no positive ICD effect on the CD spectrum was observed upon addition of the *E*-configured polyamides to the same dsDNA sequences, which indicates that they did not bind to the minor groove of the short dsDNA. Importantly, no precipitation was observed during the measurement. We hypothesize that only the (*Z*)*-*azobenzene moiety is able to induce the polyamide hairpin conformation required for binding. We also tested the interaction of the polyamides upon single base mutation in the dsDNA sequences to explore the recognition specificity. Specifically, we tested the sequence 5´-TTGT**T**-3´ for **P1**, 5´-TTGT**C**-3´ for **P2**, and 5´-TTGT**T**A-3´ for **P3** and obtained essentially similar results. Only the *Z-*isomers were able to bind, while the *E*-isomers did not show any interaction with the DNA minor groove (Figure 4).

While the complexes of *Z-***P1** and *Z-***P2** with their target DNA (**D1** and **D2**) and single-point mutation DNA (**D1’** and **D2’**) had similar ICD spectra, the complex of *Z-***P3** with the single-point mutation DNA **D3’** showed only a blue shift of the B-DNA maximum at ca. 275 nm, but no shift of the minimum at ≈250 nm, lacking this isodichroic point (cf. Figure 3C and Figure 4C). Finally, *Z-***P3** did not reach saturation with the single-point mutation DNA **D3’** at the tested concentration range, suggesting lower binding affinity compared to target dsDNA **D3**.

The maxima of the ICD signals at 325 and 333 nm were plotted against the concentration of polyamide to quantify the binding of the PA to the cognate dsDNA. The dissociation constants *K*_D_, calculated from the binding isotherms, were in the micromolar range (11.8 ± 6.0–52.9 ± 29.6 μM). The binding affinity decreased in the order **P1** > **P3** > **P2** for the target sequences and **P1** > **P2** > **P3** for the single-point mutation (Table 1).

**Table 1:** Binding constants and melting temperatures of the interaction of the polyamides with dsDNA obtained by circular dichroism.

recognition sequence (5´→3´)	*K*_D_/μM	*T*_m_/°C (DNA)^a^	*T*_m_/°C (PA/DNA)^b^	Δ*T*_m_/K	PA:DNA

	*Z-***P1**				
CATTGTCAGCCTTTTTGGCTGACAATG **D1**	11.8 ± 6.0	72.2	75.7	3.5	3.3
CATTGT**T**AGCCTTTTTGGCT**A**ACAATG **D1‘**	13.2 ± 7.8	66.5	68.6	2.1	3.3
	*Z-***P2**				
CATTGTTAGACTTTTTGTCTAACAATG **D2**	52.3 ± 21.0	65.4	67.5	2.0	3.9
CATTGT**C**AGACTTTTTGTCT**G**ACAATG **D2‘**	42.2 ± 6.3	68.7	70.1	1.4	3.6
	*Z-***P3**				
CATTGTCATGCCTTTTTGGCATGACAATG **D3**	21.3 ±11.2	72.9	76.4	3.5	3.3
CATTGT**T**ATGCCTTTTTGGCAT**A**ACAATG **D3‘**	52.9 ± 29.6	69.6	70.0	0.4	3.7

^a^*c* (dsDNA) = 8.87 μM. ^b^For the *Z-*isomers, the employed ratio is given. For specific experimental conditions see Experimental section.

The CD spectroscopic experiments indicate that the ligands *E*-**P1**–*E*-**P3** did not bind significantly to the oligonucleotide sequences **D1**–**D3**. However, considering their polyamide structure, they should at least exhibt a weak affinity toward double-stranded DNA. The fluorescent indicator displacement (FID) assay was performed with *E*-**P1** as representative ligand to examine the propensity of these derivatives to bind to DNA. The known intercalator thiazole orange (TO) was chosen as indicator because it exhibits an intense fluorescence band at 526 nm when bound to DNA, whereas it is only weakly fluorescent in solution. The displacement of TO from its DNA binding site was monitored by emission spectroscopy. The addition of 2.3 equiv of *E*-**P1** led to a displacement of 50% of TO (cf. Supporting Information File 2), which indicates only a weak binding of *E*-**P1** because as a groove binder, the latter occupies more binding sites than TO. These data indicate that *E*-**P1** does bind to DNA in general, but the affinity of this ligand was obviously too low to bind strongly enough to the duplexes **D1**–**D3**, as indicated by the lack of any significant induced circular dichroism signal.

We tested the stability of each dsDNA by determining their melting curve during thermal DNA denaturation in the absence or the presence of **P1**–**P3** under otherwise identical experimental conditions for further information on the PA/DNA interaction (see Experimental section). If a polyamide is a minor groove binder, this affects the melting temperature of the dsDNA [34,47,53–55]. The DNA double strand is stabilized by additional hydrogen bonds between ligand and oligonucleotide, and hence the melting temperature is expected to increase. This effect is of particular interest when considering the sequence selectivity of the polyamides, which should therefore also be reflected in a change of the melting temperature. The melting curves after the addition of polyamide were recorded at the highest concentration of polyamide used in the titration experiments (Table 1). Since the hyperchromicity of DNA increases the absorption of ssDNA at 260 nm compared to that of dsDNA, denaturation can be observed by CD, too. The melting temperature (*T*_m_) corresponds to the point of inflection where 50% of the DNA molecules are single-stranded. *T*_m_ depends on the number and strength of intramolecular interactions between the single strands, providing information on stabilization or destabilization of the DNA duplex in the presence of the corresponding PA. In general, the more energy needed to break the hydrogen bonds between the single strands of the duplex and the stacking interactions of the bases, the higher the melting temperature. Hence, it also increases with higher GC content. The melting temperature *T*_m_ of the dsDNA increased in the presence of the three polyamides *Z-***P1**, *Z-***P2**, and *Z-***P3**, although it is also possible that during heating, thermally induced isomerization to the *E-*isomer occurred (Table 1). The highest stabilization value of the corresponding target sequences was obtained for *Z-***P1** and *Z-***P3** (Δ*T*_m_ = 3.5 K). The lowest value was obtained for *Z-***P2** (Δ*T*_m_ = 2 K). On the other hand, for the single-point mutation, the *T*_m_ values were lower: Δ*T*_m_ = 2.1 K for *Z-***P1**, Δ*T*_m_ = 1.4 K for *Z-***P2**, and Δ*T*_m_ = 0.4 K for *Z-***P3**. These results are in agreement with the data of the titration experiments and indicate a higher sequence specificity, e.g., of *Z-***P3**. The highest binding affinity (lowest *K*_D_) was obtained for *Z-***P1**, with a slight preference for the target DNA sequence compared to the single mismatch sequence. Binding affinity as well as duplex stabilization was lowest for *Z-***P2** for both dsDNA sequences tested (Table 1). The affinity of *Z-***P3** to the target **D3** was twofold higher than to **D3’** with the single mutation sequence. However, this value is still very much different from the 29-fold selectivity observed for the γ–Im/Py polyamide analogs of **P3** obtained by DNase footprinting experiments [3]. The DNA sequence selectivity observed for *Z-***P3** was underlined by an increase of Δ*T*_m_ = 3.5 K observed for the interaction with the target sequence **D3** compared to Δ*T*_m_ = 0.4 K for **D3’**.

## Conclusion

We were able to show for the first time that designed heterocyclic polyamides **P1**–**P3**, equipped with a photoisomerizable azobenzene, bind to double-stranded DNA hairpins in the *Z-*configuration. In this case, the (*Z*)*-*azobenzene acted as a building block inducing a reverse turn. Minor groove binding was proven by CD titration experiments and melting temperature determinations. The *E*-configured azobenzene polyamides did not induce ICD assigned to the polyamide chromophores, possibly because of the formation of small soluble aggregates. *Z-***P3**, with a higher number of heterocyclic units, displayed some sequence specificity, albeit not in a range that was reported for other nonphotoswitchable polyamides. The (*Z*)*-*azobenzene linker may be sterically more demanding than the γ-aminobutyric acid linker, which would cause a dilation of the minor groove, and thus reducing the binding affinity and selectivity of the tested sequences. The previously reported analog of **P3** with γ-aminobutyric acid instead of azobenzene has been used to exert genomic effects on mRNA expression [56]. Therefore, the photoactivation of such interaction may be a future application. The incorporation of the photoswitchable 3-((3-(aminomethyl)phenyl)diazenyl)phenylacetic acid linker upon replacement of the γ-aminobutyric acid linker is a powerful strategy to obtain photoswitchable Im/Py hairpin polyamides capable of interaction with the dsDNA minor groove only in the *Z-*configuration.

## Supporting Information

File 1 Experimental section.

File 2 NMR spectra of **P1–P3** and dimers **6** and **7**.

File 3 Photoisomerization of **P1**–**P3** and TO displacement experiments.

File 4 HRMS spectra of **P1**–**P3**.
