# Supplementary material for: Photocontrolled DNA minor groove interactions of imidazole/pyrrole polyamides
Source: Beilstein J Org Chem. 2020 Jan 9;16:60–70. doi: 10.3762/bjoc.16.8 (PMC6964667; doi:10.3762/bjoc.16.8)
Supplement: File 1 — Experimental section. [file Beilstein_J_Org_Chem-16-60-s001.pdf]

BEILSTEIN JOURNAL OF ORGANIC CHEMISTRY

Supporting Information

for

Photocontrolled DNA minor groove interactions of imidazole/ pyrrole polyamides

Sabrina Müller, Jannik Paulus, Jochen Mattay, Heiko Ihmels, Veronica I. Dodero and Norbert Sewald

Beilstein J. Org. Chem. **2020,** *16,* 60–70. doi:10.3762/bjoc.16.8

Experimental section

License and Terms: This is a supporting information file under the terms of the Creative Commons Attribution License (https://creativecommons.org/licenses/by/4.0). Please note that the reuse, redistribution and reproduction in particular requires that the authors and source are credited.
The license is subject to the *Beilstein Journal of Organic Chemistry* terms and conditions: (https://www.beilstein-journals.org/bjoc)

General methods

The building blocks to synthesize the corresponding Im/Py polyamides were synthesized and purified following the established protocol of Wurtz et al. [1].

NMR spectra were acquired with a DRX-500 (Bruker, ^{1}H-NMR: 500 MHz, ^{13}*C*-NMR: 126 MHz). The assignment of the signals was performed from the one-dimensional spectra, and in some cases, two-dimensional correlation spectra (^{1}H,^{1}H COSY, ^{1}H,^{13}C HMBC, ^{1}H,^{13}C HMQC, NOESY, ROESY, TOCSY) and DEPT-135 spectra were also included for assignment. For the polyamides, the carbon signals were determined by ^{1}H,^{13}C-HMBC and ^{1}H,^{13}*C*-HMQC. Mass spectrometry was obtained using a MALDI-ToF Voyager DE (PE Biosystems) with 1.2 m flight tube, the ionization of the sample was carried out by an N_2 laser (λ-337 nm, pulse width 3 ns, repetition rate 3 Hz). DHB (2,5-dihydroxybenzoic acid) was used as the matrix. Exact mass determination (HR-ESI-MS) was performed on a MicroToF (Bruker Daltonics) with loop inlet. Mass calibration was performed immediately by quasi-internal calibration.

RP-HPLC purification. A Thermo Scientific HPLC unit was used (Spectrasystem, interface module: SN-4000, pump: P-4000, UV–vis detector: UV1000) equipped with a column Thermo Scientific Hypersil Gold (particle size 8 µm, 21.2 (ID) × 250 mm at a flow rate of 10.0 mL min^{-1}). The detection was carried out by absorption at 254 nm. The eluent was a mixture of eluent A (MeCN/H_2O/TFA 95:5:0.1 v:v:v) and eluent B (MeCN/H_2O/TFA 5:95:0.1 v:v:v). Gradient: 0%A for 5 min, from 0%A to 50%A in 55 min and from 50%A to 100% A in 5 min.

Photochemistry. Photoswitching experiments were performed in NMR tubes (Boroeco-5-7, borosilicate glass, Deutero) using RPR-100 Rayonet

photoreactor (Southern New England Ultraviolet Company) with emission wavelengths 350 nm and 420 nm (± 20 nm half-width) [2]. The operating temperature was approximately 35 °C with fan in operation. The duration of illumination is indicated in each case and the concentration of the sample also (Fig. 1-2). The corresponding ^{1}H-NMR spectra were acquired before and after illumination with UV light. For measurement of thermal photoisomerization the samples were protected from the visible light and stored when necessary at 5°C. The spectra were measured on the Bruker AV-600 and calibrated for the residual proton signals of the solvent.

Circular dichroism: CD spectra were recorded on a J-810 CD spectrometer (Jasco) using a 1 mm quartz glass cuvette (QS, Hellma) equipped with a Peltier temperature controller.

Synthesis

Fmoc-AB-OH (1): A solution of tert-butyl 3-nitrophenylacetate [3] (1.20 g, 5.06 mmol) in MeOH (25 mL) was degassed with Ar, followed by the addition of Pd/C (10%, 50 mg). The mixture was stirred overnight under H_2 atmosphere. The catalyst was removed and the filtrate concentrated. The liquid residue solidified to a grey-brown solid (1.03 g, 4.99 mmol, 99%) which was characterized as 3-aminophenylacetate. It was used for the next reaction without further purification.

^{1}H-NMR (500 MHz, $CDCl_3$): δ/ppm = 7.10 (m, 1H, C^2H); 6.66 (m, 1H, C^5H); 6.61 (m, 1H, C^4H); 6.58 (m, 1H, C^6H); 3.63 (bs, 2H, NH_2); 3.43 (s, 2H, CH_2); 1.44 (s, 9H, $C(CH_3)_3$). **^{13}*C*-NMR** (126MHz, $CDCl_3$): δ/ppm = 171.0 (CO); 146.5 (C^3);

135.8 (C^1); 129.3 (C^5); 119.5 (C^6); 115.9 (C^2); 113.7 (C^4); 80.7 (**C**(CH_3)$_3$); 42.6 (CH_2); 28.1 (C(**C**H_3)$_3$).

A solution of Oxone® (6.09 g, 9.91 mmol) in H_2O (80 mL) was added to a solution of *tert*-butyl 3-aminophenylacetate (1.03 g, 4.95 mmol) in CH_2Cl_2 (20 mL). The mixture was stirred vigorously for 4 h at rt The phases were separated and the aqueous phase was extracted with CH_2Cl_2 (2 × 25 mL). The combined organic phases were washed with 1 N HCl, sat. aqueous $NaHCO_3$ solution and sat. NaCl solution followed by drying over Na_2SO_4 and concentration in vacuum. The residue was purified by column chromatography (PE/EtOAc 4:1) and the resulting grass-green liquid (0.75 g) was dissolved in glacial acetic acid (10 mL) and a solution of 9*H*-fluoren-9-yl)methyl(3-aminobenzyl)carbamate [4] (1.17 g, 3.41 mmol) in glacial acetic acid (15 mL) was added. The reaction mixture was stirred for 20 h at rt under exclusion of light. After removal of the solvent, the residue was purified by column chromatography (PE/EtOAc 4:1), giving azobenzene derivative **1** as an orange solid (1.30 g, 2.37 mmol, 48% over two steps). The NMR signals correlate with those described in the literature [3]. **^{1}H-NMR** (500 MHz, $CDCl_3$): δ/ppm = 7.85 – 7.80 (m, 4H, AB-C^2H, AB-$C^{2'}$H, AB-C^6H, AB-$C^{6'}$H,); 7.77 (d, ^3J=7.5Hz, 2H, Fmoc-C^{ar}H); 7.61 (d, ^3J=7.4Hz, 2H, Fmoc-C^{ar}H);7.50–7.37 (m, 6H, Fmoc-C^{ar}H, AB-C^{ar}H); 7.33 – 7.28 (m, 2H, Fmoc-C^{ar}H); 5.20 (m, 1H, Fmoc-H^N); 4.51 – 4.48 (m, 4H, Fmoc-CH_2, AB-CH_2-H^N), 4.25 (t, ^3J=6.8 Hz, 1H, Fmoc-CH); 3.64 (s, 2H, AB-CH_2-CO); 1.45 (s, 9H, C(CH_3)$_3$). **^{13}*C*-NMR** (126MHz, $CDCl_3$): δ/ppm = 171.2 (*t*-BuO-CO); 156.5 (Fmoc-CO); 152.9,152.7 (AB-C^1, AB-$C^{1'}$); 143.9, 141.3 (Fmoc-C^{ar}); 139.6, 135.8 (AB-C^3, AB-$C^{3'}$); 132.0, 130.0, 129.5, 129.2 (AB-C^{ar}); 127.7, 127.1, 125.0 (Fmoc-C^{ar}H); 123.5, 122.4, 121.9, 121.4 (AB-C^{ar}); 120.0 (Fmoc-C^{ar}H); 81.2

($\mathbf{C}(CH_3)_3$); 66.8 (Fmoc-CH_2); 47.3 (Fmoc-CH); 44.9 (AB-CH_2-H^N); 42.4 (AB-$\mathbf{C}H_2$-CO); 28.1 (C($\mathbf{C}H_3)_3$).

Fmoc-AB-Im-Ot-Bu (6): A slurry of Pd/C in THF (10%, 140 mg) was added to a degassed solution of 1-methyl-4-nitroimidazole-2-*tert*-butylester [5] (1.17 g, 5.15 mmol) in THF (55 mL) and H_2 was passed through the reaction mixture. The reaction was monitored by TLC (EtOAc). After 5 h, the reaction mixture was filtered and the filtrate was concentrated. The resulting amine (**4**, Scheme 2A) was dissolved in THF (50 mL) and treated with a solution of Fmoc-AB-OH acid (**1**, Scheme 2A) (1.00 g, 2.03 mmol) and DCC (726 mg, 2.23 mmol) in THF (50 mL) previously activated in the dark for 5 min. The reaction mixture was stirred for 15 h in the dark at room temperature. The product mixture was filtered and the filtrate was concentrated, followed by dilution in EtOAc. The solution was washed successively with 1 N HCl and a saturated solution of $NaHCO_3$, dried over Na_2SO_4 and concentrated. Column chromatographic purification of the crude product (PE/EtOAc 7: 3) gave compound (**6**) as an orange solid (0.56 g, 0.83 mmol, 41%). $\mathbf{R_f}$ = 0.34 (PE/EtOAc) 1:1). **^{1}H-NMR** (500 MHz, $CDCl_3$): δ/ppm = 8.30 (s, 1H, Im-H^N); 7.86 – 7.83 (m, 3H, AB-C^{ar}H); 7.80 (s, 1H, AB-C^{ar}H); 7.76 (d, ^3J=7.5Hz, 2H, Fmoc-C^{ar}H); 7.61 (d, ^3J=7.3Hz, 2H, Fmoc-C^{ar}H); 7.51 – 7.48 (m, 3H, AB-C^{ar}H, Im-C^5H); 7.41 – 7.38 (m, 4H, AB-C^{ar}H, Fmoc-C^{ar}H); 7.33 – 7.28 (m, 2H, Fmoc-C^{ar}H); 5.28 (m, 1H, AB-CH_2-$\mathbf{H^N}$); 4.51 (d, ^3J=6.0 Hz, 2H, AB-C$\mathbf{H_2}$-H^N); 4.48 (d, ^3J =6.9 Hz, 2H, Fmoc-CH_2); 4.25 (t, ^3J=6.9 Hz, 1H, Fmoc-CH); 3.92 (s, 3H, Im-CH_3); 3.80 (s, 2H, AB-CH_2-CO); 1.57 (s, 9H, C($CH_3)_3$). **^{13}*C*-NMR** (151 MHz, $CDCl_3$): δ / ppm = 167.8 (AB-CO); 158.0 (Fmoc-CO); 156.5 (Im-CO); 152.9, 152.8 (AB-C^1, AB-$C^{1'}$); 143.9, 141.3 (Fmoc-C^{ar}); 139.7 (AB-$C^{3'}$); 136.6 (AB-C^3);135.1 (Im-C^4);

132.6 (AB-C^{ar}); 132.1 (Im-C^2); 130.2 (AB-C^{ar}); 129.9, 129.5 (AB-C^6, AB-$C^{6'}$); 127.7, 127.1, 125.1 (Fmoc-C^{ar}H); 123.5, 122.7, 122.6, 121.4 (AB-C^{ar}); 120.0 (Fmoc-C^{ar}H); 114.8 (Im-C^5); 83.0 (**C**(CH_3)$_3$); 66.8 (Fmoc-CH_2); 47.3 (Fmoc-CH); 44.9 (AB-CH_2-H^N); 43.8 (AB-**C**H_2-CO); 36.2 (Im-CH_3); 28.2 (C(**C**H_3)$_3$); **ESI-MS** for $[C_{39}H_{38}N_6O_5+Na]^+$ calc. 693.2796, obs. 693.2785.

Fmoc-AB-Im-OH (**7**): Boron trifluoride (48% BF_3, 2.1 mL, 8.34 mmol) was added at 0 °C to a solution of the ester **5** (560 mg, 0.83 mmol) in abs. CH_2Cl_2 (15 mL) and stirred at rt (TLC control: PE/EtOAc 1:2). After 12 h, the solution was mixed with 2 M aqueous HCl (35 mL) and stirred at rt for a few minutes. After that, the resulting solid was filtered off, washed with little H_2O and dried to give the acid **6** as an orange solid (374 mg, 0.61 mmol, 73%). **RP-HPLC: t_R** = 4.34 min (Gradient: 0%A to 100% A in 5 min, 35 °C (A: MeCN/H_2O/TFA 95:5:0.1 (*v*:*v*:*v*); B:MeCN/H_2O/TFA 5:95:0.1 (*v*:*v*:*v*), column Thermo Scientific Hypersil Gold, 3 µm, 2.1(D)× 150mm). **^{1}H-NMR** (500 MHz, DMSO-d_6): δ/ppm = 10.92 (s, 1H, Im-H^N); 7.99 (t, ^3J=6.1 Hz, 1H, AB-CH_2-**H**^{N}); 7.88 (d, ^3J= 7.5 Hz, 2H, Fmoc-C^{ar}H); 7.85 (s, 1H, AB-C^2H); 7.78 – 7.76 (m, 3H, AB-$C^{2'}$H, AB-C^{ar}H); 7.70 (d, ^3J= 7.5 Hz, 2H, Fmoc-C^{ar}H); 7.56 – 7.50 (m, 3H, AB-C^{ar}H); 7.47 (s, 1H, Im-C^5H); 7.42 (d, J= 7.9 Hz, 1H, AB-C^{ar}H); 7.42 – 7.37 (m, 2H, Fmoc-C^{ar}H); 7.33 – 7.28 (m, 2H, Fmoc-C^{ar}H); 4.36 (d, ^3J = 6.8 Hz, 2H, Fmoc-CH_2); 4.30 (d, ^3J= 6.0 Hz, 2H, AB-C**H$_2$**-H^N); 4.24 (t, ^3J = 6.8 Hz, 1H, Fmoc-CH); 3.87 (s, 3H, Im-CH_3); 3.74 (s, 2H, AB-CH_2 CO). **^{13}*C*-NMR** (126 MHz, DMSO-d_6): δ/ppm = 167.5 (COOH); 160.0 (AB-CO); 156.4 (Fmoc-CO); 152.0, 151.9 (AB-C^1, AB-$C^{1'}$); 143.9 (Fmoc-C^{ar}); 141.4 (AB-C^3); 140.7 (Fmoc-C^{ar}); 137.5 (AB-$C^{3'}$); 137.0 (Im-C^4); 132.3 (AB-C^{ar}); 131.8 (Im-C^2); 130.2 (AB-C^{ar}); 129.4, 129.4 (AB-C^6, AB-$C^{6'}$); 127.6, 127.0, 125.1 (Fmoc-C^{ar}H); 122.5, 121.6, 121.5, 120.5

(AB-C^{ar}); 120.1 (Fmoc-C^{ar}H); 114.6 (Im-C^5); 65.4 (Fmoc-CH_2); 46.8 (Fmoc-CH); 43.5 (AB-CH_2-H^N); 41.9 (AB-**C**H_2-CO); 35.5 (Im-CH_3). **ESI-MS**: $[C_{35}H_{30}N_6O_5+Na]^+$, calc. 637.21699, obs. 637.21725.

Solid phase synthesis

Reaction monitoring. To check the progress of the reaction, some resin beads were taken, mixed with 30 µL of 95% TFA solution and shaken for a few seconds, while the beads turn red. The supernatant solution was removed and mixed 1:1 with H_2O. 1 µL of the aqueous solution was mixed with 1 µL of DHB matrix on the target, dried and the mass spectrum was measured by MALDI–ToF–MS. If necessary, the deprotection was repeated.

Fmoc deprotection. The amino protecting group Fmoc was cleaved after each coupling step to allow the coupling of the next amino acid. For this, the resin was swollen in DMF and then suspended three times for 15 minutes in a 20% piperidine solution in DMF. The resin was filtered off and washed twice with CH_2Cl_2, DMF and again CH_2Cl_2.

Coupling reactions. The resin was swollen in DMF. The amino acid (3 equiv) was dissolved in DMF containing DIPEA (9 equiv) and the coupling reagent (HBTU or PyBOP, 3 equiv) was added for pre-activation. After 5 minutes the solution was added to the resin, and the suspension was mixed for 1.5 hours by shaking. The resin was then filtered off with suction and washed twice with CH_2Cl_2, DMF and again CH_2Cl_2. The completeness of the reaction was checked with MALDI–ToF–MS. If necessary, the coupling reaction was repeated.

Cleavage from the resin. After swelling in CH_2Cl_2, the acid-labile 2 chlorotrityl resin containing the polyamide was mixed with a 2.5% solution of

CH_2Cl_2/TFA/TIS 95:2.5:2.5 (v/v/v), mixed for 5 minutes and filtered off. This was repeated until the discoloration of the filtrate was complete. The combined filtrates were evaporated and the oily residue was treated with a mixture H_2O:MeCN and lyophilized.

Im–Py–Py–AB–Im–Py–Py–β–Dp (**P1**): Polyamide **P1** was assembled on 1.00 g of Fmoc-β-alanine-loaded 2 chlorotrityl resin (loading: 0.220 mmol g^{-1}) as described above using the building blocks 4-(fluorenylmethoxycarbonylamino)-1-methylpyrrole-2-carboxylic acid (**2a**), 1-methylimidazole-2-carboxylic acid (**3b**), Fmoc-AB-Im-OH (**7**) and the coupling reagent PyBOP. It was cleaved from the resin and conjugated in solution with *N*,*N*-dimethylaminopropylamine (coupling reagent: PyBOP). Purification was carried out by preparative RP HPLC and the target polyamide was obtained as TFA salt (44 mg, 35 µmol, 16%). **RP-HPLC:** t_R = 9.74 min (Gradient: 0% A to 100% A in 12 min, 35°C (A: MeCN/H_2O/TFA 95:5:0.1 (*v*/*v*/*v*); B: MeCN/H_2O/TFA 5:95:0.1 (*v*/*v*/*v*), column Thermo Scientific Hypersil Gold (3 µm, 2.1(D)× 150mm). **^{1}H-NMR** (600MHz, DMSO-d_6): δ/ppm = 10.57 (s, 1H, Im^4-H^N); 10.45 (s, 1H, Py^2-H^N); 10.02 (s, 1H, Py^5-H^N); 9.94 (s, 1H, Py^3-H^N); 9.98 (s, 1H, Py^6-H^N); 9.33 (bs, 1H, $(CH_3)_2N\mathbf{H}^+$); 8.72 (t, 1H, ^3J=6.0 Hz, AB-CH_2-H^N); 8.05 (t, 1H, ^3J= 5.7 Hz, Dp-H^N); 8.02 (t, 1H, ^3J=5.4 Hz, β-H^N); 7.88 (s, 1H, AB-$C^{2'}$H); 7.84 (s, 1H, AB-C^2H); 7.81 – 7.77 (m, 2H, AB-$C^{6'}$H, AB-C^6H); 7.58 – 7.54 (m, 2H, AB-$C^{5'}$H, AB-C^5H); 7.53 – 7.51 (m, 2H, AB-$C^{4'}$H, AB-C^4H); 7.44 (s, 1H, Im^4-CH); 7.39 (s, 1H, Im^1-C^5H); 7.28 (d, 1H, ^4J=1.6Hz, Py^2-C^5H); 7.25 (d, 1H, ^4J= 1.5Hz, Py^5-C^5H); 7.23 (d, 1H, ^4J=1.6 Hz, Py^3-C^5H); 7.16 (s, 2H, Py^6-C^5H, Py^2-C^3H); 7.14 (d, 1H, ^4J=1.5 Hz, Py^5-C^3H); 7.05 (s, 1H, Im^1-C^4H); 7.01 (d, 1H, ^4J = 1.6 Hz, Py^3-C^3H); 6.88 (d, 1H, ^4J=1.6 Hz, Py^6-C^3H); 4.51 (d, 2H, ^3J=5.8Hz, AB-C$\mathbf{H_2}$-H^N); 3.99 (s, 3H, Im^1-CH_3); 3.93

(s, 3H, Im^4-CH_3); 3.84 (s, 6H, Py^6-CH_3, Py^3-CH_3); 3.83 (s, 3H, Py^2-CH_3); 3.80 (s, 3H, Py^5-CH_3); 3.79 (s, 2H, AB-CH_2-CO); 3.39 (dt, 2H, ^3J =6.9, 6.9Hz, β-$C^{\beta}H_2$); 3.12 (dt, 2H, ^3J = 6.3, 6.4Hz, Dp-C**H_2**-H^N); 3.03 – 2.99 (m, 2H, $(CH_3)_2NH^+$C**H_2**); 2.75 (s, 3H, C**H_3**NH^+); 2.74 (s, 3H, C**H_3**NH^+); 2.35 (t, 2H, ^3J = 7.0 Hz, β-$C^{\alpha}H_2$); 1.74 (m, 2H, Dp-C**H_2**CH_2-H^N). **$^{13}C\{^1H\}$-NMR** (151 MHz, DMSO-d_6): δ / ppm = 170.9 (β-CO); 167.6 (AB-CH_2-**C**O); 161.2 (Py^3-CO); 161.1 (Py^6-CO); 158.9 (Py^5-CO); 158.3 (Py^2-CO); 155.8 (Im^1-CO); 155.6 (Py^4-CO); 152.4 (AB-C^1, AB-$C^{1'}$); 141.8 (AB-C^3); 138.6 (Im^1-C^2); 137.5 (AB-$C^{3'}$);135.8 (Im^4-C^4); 134.1 (Im^4-C^2); 132.2 (AB-$C^{4'}$); 130.8 (AB-$C^{5'}$); 129.6 (AB-C^4); 129.4 (AB-C^5); 126.9 (Im^1-C^4); 126.3 (Im^1-C^5); 123.0 (Py^3-C^2); 122.9 (Py^6-C^2); 122.7 (Py^5-C^2); 122.5 (AB-$C^{2'}$); 122.4 (Py^2-C^2); 122.3 (Py^6-C^4); 122.1 (Py^3-C^4); 121.6 (Py^2-C^4); 121.4 (AB-C^6, AB-$C^{6'}$);120.9 (AB-C^2); 120.9 (Py^5-C^4); 118.6 (Py^2-C^5); 118.5 (Py^5-C^5); 118.2 (Py^3-C^5); 117.8 (Py^6-C^5); 114.1 (Im^4-C^5); 104.8 (Py^5-C^3, Py^2-C^3); 104.5 (Py^3-C^3); 104.2 (Py^6-C^3); 54.7 ($(CH_3)_2NH^+$**C**H_2); 42.3 (CH_3NH^+); 41.9 (AB-**C**H_2-CO); 41.7 (AB-CH_2-H^N); 36.2 (Py^5-CH_3, Py^2-CH_3); 36.1 (Py^3-CH_3); 36.0 (Py^6-CH_3); 35.6 (β-C^{α}); 35.5 (Dp-CH_2-H^N); 35.4 (β-C^{β}); 35.1 (Im^1-CH_3); 34.9 (Im^4-CH_3); 24.5 (Dp-**C**H_2CH_2-H^N). **HR-MS:** $[C_{57}H_{65}N_{19}O_8+H]^+$ calc. 1144.5336, obs. 1144.5309

Im–Py–Py–AB–Py–Py–Py–β–Dp (P2): The polyamide P2 was synthesized on 1.00 g of Fmoc-β-alanine-loaded 2-chlorotrityl resin (loading: 0.233 mmol g^{-1}) as described above using the building blocks 4-(fluorenylmethoxycarbonylamino)-1-methylpyrrole-2-carboxylic acid (**2a**), 1-methyl-imidazole-2-carboxylic acid (**3b**), and Fmoc-AB-OH (**1**) and the coupling reagent HBTU. It was cleaved from the resin and conjugated with *N*,*N*-dimethylaminopropylamine in solution (coupling reagent: HBTU). Purification

was carried out by preparative RP-HPLC and the target polyamide was obtained as TFA salt (6 mg, 5 µmol, 2%). **RP-HPLC:** t_R = 9.53 min (Gradient: 0% A to 100% A in 12 min, 35°C (A: MeCN/H_2O/TFA 95:5:0.1 (*v/v/v*); B: MeCN/H_2O/TFA 5:95:0.1 (*v/v/v*), column Thermo Scientific Hypersil Gold (3 µm, 2.1(D)× 150mm). **HR-MS:** $[C_{58}H_{66}N_{18}O_8+Na+H]^+$ calc. 1143.5384, obs. 1143.5357. **^{1}H-NMR** (600 MHz, DMSO-d_6): δ/ppm = 10.48 (s, 1H, Py^2-H^N); 10.17 (s, 1H, Py^4-H^N); 9.94 (s, 1H, Py^3-H^N); 9.89 (s, 1H, Py^5-H^N); 9.87 (s, 1H, Py^6-H^N); 9.62 (bs, 1H, $(CH_3)_2N\mathbf{H}^+$); 8.71 (t, ^3J= 6.2 Hz, 1H, AB-CH_2-$\mathbf{H}^N$); 8.05 (t, ^3J = 5.9 Hz, 1H, Dp-H^N); 8.01 (t, ^3J= 5.7 Hz, 1H, β-H^N); 7.86 (s, 1H, AB-$C^{2'}$H); 7.84 (s, 1H, AB-C^2H); 7.81 – 7.76 (m, 2H, AB-C^6H, AB-$C^{6'}$H);7.59 – 7.50 (m, 4H, AB-C^4H, AB-$C^{4'}$H, AB-C^5H, AB-$C^{5'}$H); 7.41 (s, 1H, Im^1-C^5H); 7.29 (d, ^4J = 1.9 Hz, 1H, Py^2-C^5H); 7.23 (d, ^4J = 1.9 Hz, 1H, Py^5-C^5H); 7.21 (d, ^4J = *1*.9 Hz, 1H, Py^3-C^5H); 7.18 – 7.15 (m, 3H, Py^4-C^5H, Py^6-C^5H, Py^2-C^3H); 7.08 (s, 1H, Im^1-C^4H); 7.04 (d, ^4J = 2.0 Hz, 1H, Py^5-C^3H); 7.01 (d, ^4J= 1.9 Hz, 1H, Py^3-C^3H); 6.92 (d, ^4J= 2.0 Hz, 1H, Py^4-C^3H); 6.87 (d, ^4J = 1.9 Hz, 1H, Py^6-C^3H); 4.51 (d, ^3J = 6.2 Hz, 2H, AB-C$\mathbf{H_2}$-H^N); 3.99 (s, 3H, Im^1-CH_3); 3.85 (s, 3H, Py^2-CH_3); 3.84 (s, 3H, Py^5-CH_3); 3.83 (s, 3H, Py^3-CH_3); 3.82 (s, 3H, Py^4-CH_3); 3.80 (s, 3H, Py^6-CH_3); 3.72 (s, 2H, AB-CH_2-CO); 3.42 – 3.34 (m, 2H, β-$C^\beta H_2$); 3.15 – 3.06 (m, 2H, Dp-C$\mathbf{H_2}$-H^N); 3.04 – 2.96 (m, 2H, $(CH_3)_2NH^+C\mathbf{H_2}$); 2.73 (s, 3H, C$\mathbf{H_3}$$NH^+$); 2.72 (s, 3H, C$\mathbf{H_3}$$NH^+$); 2.35 (t, ^3J = 7.2 Hz, 2H, β-$C^\alpha H_2$); 1.81 – 1.70 (m, 2H, Dp-C$\mathbf{H_2}$$CH_2$-$H^N$). **^{13}C{^{1}H}-NMR** (151 MHz, DMSO-d6): δ / ppm = 171.3 (β-CO); 167.7 (AB-CH_2-**C**O);161.7 (Py^3-CO); 161.5 (Py^6-CO); 158.9 (Py^5-CO); 158.7 (Py^4-CO);158.2 (Py^2-CO); 156.1 (Im^1-CO); 152.4 (AB-C^1, AB-$C^{1'}$); 142.3 (AB-C^3); 139.0 (Im^1-C^2); 138.3 (AB-$C^{3'}$);132.5 (AB $C^{4'}$); 130.7 (AB-C^4); 129.9 (AB-C^5, AB-$C^{5'}$); 127.0 (Im^1-C^4); 126.8 (Im^1-C^5);123.5 (Py^2-C^2); 123.2 (Py^6-C^2,

Py^4-C^2); 123.1 (Py^5-C^2); 123.0 (Py^3-C^2, AB-C$^{2'}$); 122.6 (Py^3-C^4); 122.5 (Py^6-C^4); 122.4 (Py^5-C^4); 122.3 (Py^4-C^4); 121.8 (Py^2-C^4, AB-C^6, AB-C$^{6'}$); 121.3 (AB-C^2);119.1 (Py^2-C^5); 118.9 (Py^5-C^5); 118.8 (Py^4-C^5); 118.7 (Py^3-C^5); 118.4 (Py^6-C^5); 105.3 (Py^2-C^3); 105.1 (Py^5-C^3); 105.0 (Py^3-C^3); 104.7 (Py^6-C^3); 104.6 (Py^4-C^3); 55.1 ((CH_3)$_2$NH$^+$**C**H$_2$); 42.8 (AB-**C**H$_2$-CO); 42.6 (CH$_3$NH$^+$); 42.1 (AB-**C**H$_2$-H^N); 36.6 (Py^5-CH$_3$, Py^4-CH$_3$, Py^3-CH$_3$, Py^2-CH$_3$); 36.5 (Py^6-CH$_3$); 36.1 (β-C$^\alpha$); 35.9 (β-C$^\beta$, Dp-CH$_2$-H^N); 35.6 (Im^1-CH$_3$); 24.9 (Dp-**C**H$_2$CH$_2$-H^N).

Im–Py–Py–Py–AB–Py–Im–Py–Py–β–Dp (P3)

Polyamide **P3** was synthesized on 1.00 g of Fmoc-β-alanine-loaded 2-chlorotrityl resin (loading: 0.220 mmol g^{-1}) as described above using the building blocks 4-(fluorenylmethoxycarbonylamino)-1-methylpyrrole-2-carboxylic acid (**2a**), 1-methylimidazole-2-carboxylic acid (**3b**), Fmoc-AB-OH (**1**), Fmoc-Py-Im-OH (**5**) [1], and the coupling reagent PyBOP. It was cleaved from the resin and conjugated with *N*,*N*-dimethylaminopropylamine in solution using the coupling reagent PyBOP. Purification was carried out by preparative RP HPLC and the desired polyamide was obtained as TFA salt (18 mg, 12 µmol, 6%). **RP HPLC:** t_R = 9.99 min (Gradient: 0% A to 100% A in 12 min, 35 °C (A: MeCN/H_2O/TFA 95:5:0.1 (*v*/*v*/*v*); B:MeCN/H_2O/TFA 5:95:0.1 (*v*/*v*/*v*), column Thermo Scientific Hypersil Gold, 3 µm, 2.1×150mm). **^{1}H-NMR** (600 MHz, DMSO-d_6): δ/ppm = 10.44 (s, 1H, Py^2-H^N); 10.24 (s, 1H, Im^6-H^N); 10.19 (s, 1H, Py^5-H^N); 9.99 (s, 1H, Py^3-H^N); 9.95 (s, 1H, Py^7-H^N); 9.92 (s, 1H, Py^4-H^N); 9.90 (s, 1H, Py^8-H^N); 9.43 (bs, 1H, (CH_3)$_2$N**H**$^+$); 8.72 (t, ^3J = 5.3 Hz, 1H, AB-CH$_2$-**H**^{N}); 8.08 – 8.01 (m, 2H, β-H^N, Dp-H^N); 7.86 (s, 1H, AB-C$^{2'}$H); 7.84 (s, 1H, AB-C^{2}H); 7.81 – 7.76 (m, 2H, AB-C^{6}H, AB-C$^{6'}$H); 7.60 – 7.48 (m, 5H, Im^6-C^{5}H, AB-C$^{4'}$H, AB-C^{4}H, AB-C$^{5'}$H, AB-C^{5}H); 7.40 (s, 1H, Im^1-C^{5}H); 7.33 – 7.21 (m, 5H,

Py^{2}-C^{5}H, Py^{3}-C^{5}H, Py^{4}-C^{5}H, Py^{5}-C^{5}H, Py^{7}-C^{5}H); 7.17 (s, 2H, Py^{2}-C^{3}H, Py^{8}-C^{5}H); 7.15 (s, 1H, Py^{7}-C^{3}H); 7.08 – 7.03 (m, 2H, Im^{1}-C^{4}H, Py^{3}-C^{3}H); 7.01 (s, 1H, Py^{4}-C^{3}H); 6.98 (s, 1H, Py^{5}-C^{3}H); 6.88 (s, 1H, Py^{8}-C^{3}H); 4.51 (d, ^{3}J = 5.4 Hz, 2H, AB-C**H$_{2}$**-H^{N}); 4.00 – 3.98 (s, 6H, Im^{6}-CH$_{3}$, Im^{1}-CH$_{3}$); 3.97 – 3.81 (m, 18H, Py^{2}-CH$_{3}$, Py^{3}-CH$_{3}$, Py^{4}-CH$_{3}$, Py^{5}-CH$_{3}$, Py^{7}-CH$_{3}$, Py^{8}-CH$_{3}$); 3.73 (s, 2H, AB-CH$_{2}$-CO); 3.40 – 3.37 (m, 2H, β-C^{β}H$_{2}$); 3.13 – 3.10 (m, 2H, Dp-C**H$_{2}$**-H^{N}); 3.02 – 2.98 (m, 2H, (CH$_{3}$)$_{2}$NH^{+}C**H$_{2}$**); 2.74 (s, 3H, C**H$_{3}$**NH^{+}); 2.73 (s, 3H, C**H$_{3}$**NH^{+}); 2.37 (t, ^{3}J= 7.0Hz, 2H, β-C^{α}H$_{2}$); 1.77 – 1.72 (m, 2H, Dp-C**H$_{2}$**CH$_{2}$-H^{N}). **^{13}C{^{1}H}-NMR** (151 MHz, DMSO-d_{6}): δ / ppm = 171.4 (β-CO); 167.5 (AB-CH$_{2}$-**C**O); 161.6 (Py^{8}-CO); 161.7 (Py^{4}-CO); 159.1 (Py^{5}-CO); 158.8 (Py^{7}-CO, Im^{6}-CO, Py^{3}-CO); 156.4 (Im^{1}-CO); 156.2 (Py^{2}-CO); 152.3 (AB-C^{1}, AB-C$^{1´}$); 142.3 (AB-C^{3}); 139.1 (Im^{1}-C^{2}); 138.4 (AB-C$^{3´}$); 136.5 (Im^{6}-C^{4}); 134.4 (Im^{6}-C^{2}); 132.6 (AB-C$^{4´}$); 130.8 (AB-C^{4}); 129.8 (AB-C^{5}, AB-C$^{5´}$); 127.3 (Im^{1}-C^{4}); 126.8 (Im^{1}-C^{5}); 123.5 (Py^{7}-C^{2} , Py^{2}-C^{2}); 123.4 (Py^{5}-C^{2}); 123.3 (Py^{8}-C^{2}); 123.2 (Py^{3}-C^{2}); 123.0 (AB-C$^{2´}$, Py^{4}-C^{2}); 122.7 (Py^{5}-C^{4}); 122.5 (Py^{8}-C^{4}, Py^{7}-C^{4}); 122.6 (Py^{4}-C^{4});122.3 (Py^{2}-C^{4}); 122.1 (Py^{3}-C^{4}); 121.8 (AB-C^{6}, AB-C$^{6´}$); 121.4 (AB-C^{2}); 119.6 (Py^{5}-C^{5});119.1 (Py^{2}-C^{5}); 118.9 (Py^{3}-C^{5}); 118.8 (Py^{7}-C^{5}); 118.7 (Py^{4}-C^{5}); 118.4 (Py^{8}-C^{5}); 115.3 (Im^{6}-C^{5}); 105.4 (Py^{2}-C^{3}); 105.2 (Py^{5}-C^{3}, Py^{3}-C^{3}); 105.1 (Py^{7}-C^{3}, Py^{4}-C^{3}); 104.9 (Py^{8}-C^{3});55.3 ((CH$_{3}$)$_{2}$NH^{+}**CH$_{2}$**); 42.9 (AB-**C**H$_{2}$-CO); 42.7 (CH$_{3}$NH^{+}); 42.2 (AB-CH$_{2}$-H^{N}); 36.5 (Py^{8}-CH$_{3}$, Py^{7}-CH$_{3}$, Py^{5}-CH$_{3}$, Py^{4}-CH$_{3}$, Py^{3}-CH$_{3}$, Py^{2}-CH$_{3}$); 36.1 (β-C^{α}); 35.9 (Dp-CH$_{2}$-H^{N}, β-C^{β});35.6 (Im^{6}-CH$_{3}$, Im^{1}-CH$_{3}$); 26.4 (Dp-**C**H$_{2}$CH$_{2}$-H^{N}). **HR-MS:** [$C_{69}H_{77}N_{23}O_{10}$+H]$^{+}$ calc. 1388.62965, obs. 1388.63097.

Interaction of the polyamides with DNA: annealing of dsDNA: From the DNA obtained as a lyophilised powder, an approximately 1 mM solution in MilliQ

H_2O was prepared, and the exact concentration was determined by UV–vis using a NanoDrop™ 1000 UV–vis spectrophotometer (Thermo Scientific). From this stock solution, solutions (c = 50 μM) in sodium dimethyl arsinate buffer (10 mM sodium dimethyl arsinate, 10 mM KCl, 10 mM $MgCl_2$, 5 mM $CaCl_2$, pH 6.9 [47]) were prepared and heated to 95 °C. By slowly cooling to room temperature, the hybridised hairpin DNA was obtained and employed for CD measurements.

CD titrations. Solutions of the photoswitchable polyamides in the thermal equilibrium (predominantly *E*) and also in the photochemically obtained metastable state (predominantly *Z*-form) were used. The *E*-isomers of **P1**–**P3** were soluble in the employed buffer, while the solubility of the *Z*-isomer was limited and up to 6% DMSO had to be added as the cosolvent to the buffer. The titrations were performed at 20 °C by adding between 1–8 μL of a 0.375–0.934 mM polyamide solution (0–4.8 equiv) to a 10 μM solution of dsDNA in sodium dimethyl arsinate buffer (pH 6.9). After each addition, the CD spectrum was recorded at a rate of 100 nm min^{-1} in the range 240–380 nm. The CD spectra were normalized to equimolar DNA concentrations (10 μM) and smoothened (Savitzky-Golay, 197 points). Dissociation constants were determined using the software GraphPad Prism (Graphpad Software, Inc.).

DNA melting curves. DNA melting curves were recorded at 260 nm with one data point per 0.2 °C. The experiments were carried out in a 1 mm quartz glass cuvette (QS, Hellma). The samples consisting of dsDNA solution (9.40 μM) in sodium dimethyl arsinate buffer (pH 6.9) / 6% DMSO or dsDNA and polyamide (for each ratio see Table 1) in sodium dimethyl arsinate buffer / 6% DMSO were heated at a rate of 0.4 K min^{-1} up to 95 °C. The melting temperature (T_m) for

each interaction was determined by nonlinear regression using the software GraphPad Prism (GraphPad Software, Inc.).

References:

1. Wetzler, M.; Wemmer, D. E. *Org. Lett.* **2010**, *12*, 3488–3490.
2. Papadakis, R.; Li, H.; Bergman, J.; Lundstedt, A.; Jorner, K.; Ayub, R.; Haldar, S.; Jahn, B. O.; Denisova, A.; Zietz, B.; Lindh, R.; Sanyal, B.; Grennberg, H.; Leifer, K.; Ottosson, H. *Nat. Comm.* **2016**, *7*, 12962.
3. Brieke, C.; Rohrbach, F.; Gottschalk, A.; Mayer, G.; Heckel, A. *Angew. Chem. Int. Ed.* **2012**, *34*, 8446-8476.
4. Wurtz, N. R.; Turner, J. M.; Baird, E. E.; Dervan, P. B. *Org. Lett.* **2001**, *3*, 1201–1203.
5. Aemissegger, A.; Kräutler, V.; van Gunsteren, W. F.; Hilvert, D. *J. Am. Chem. Soc.* **2005**, *127*, 2929–2936.
