# Supplementary material for: Photocontrolled DNA minor groove interactions of imidazole/pyrrole polyamides
Source: Beilstein J Org Chem. 2020 Jan 9;16:60–70. doi: 10.3762/bjoc.16.8 (PMC6964667; doi:10.3762/bjoc.16.8)
Supplement: File 2 — NMR spectra of P1–P3 and dimers 6 and 7. [file Beilstein_J_Org_Chem-16-60-s002.pdf]

BEILSTEIN JOURNAL OF ORGANIC CHEMISTRY

# Supporting Information

for

# Photocontrolled DNA minor groove interactions of imidazole/ pyrrole polyamides

Sabrina Müller, Jannik Paulus, Jochen Mattay, Heiko Ihmels, Veronica I. Dodero and Norbert Sewald

*Beilstein J. Org. Chem.* **2020,** *16,* 60–70. doi:10.3762/bjoc.16.8

# NMR spectra of P1–P3 and dimers 6 and 7

License and Terms: This is a supporting information file under the terms of the Creative Commons Attribution License (https://creativecommons.org/licenses/by/4.0). Please note that the reuse, redistribution and reproduction in particular requires that the authors and source are credited. The license is subject to the *Beilstein Journal of Organic Chemistry* terms and conditions: (https://www.beilstein-journals.org/bjoc)

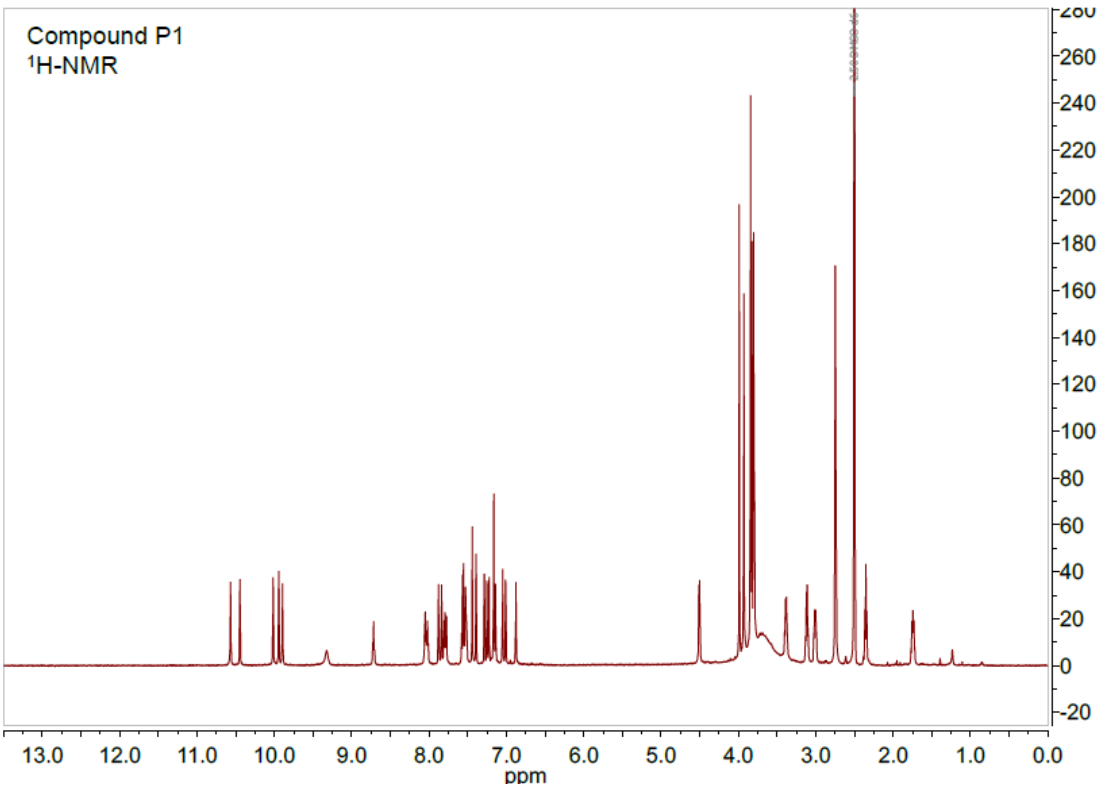
Compound P1
$^1$H-NMR
ppm

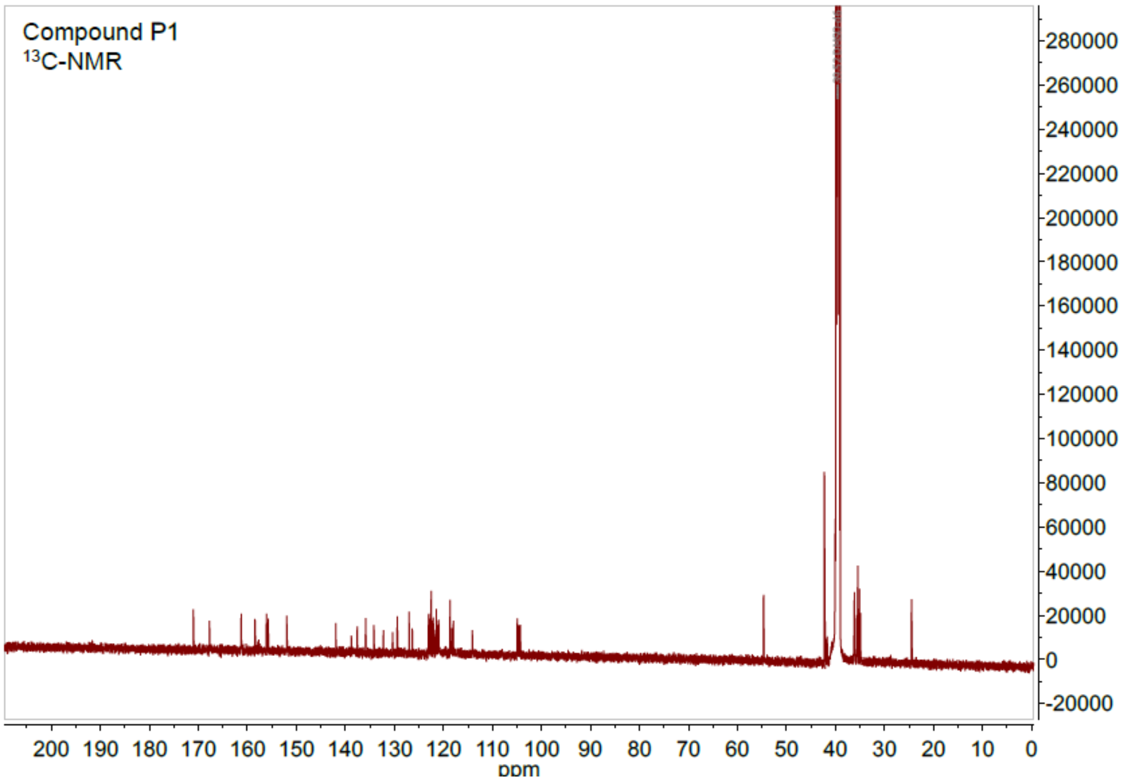
Compound P1
$^{13}$C-NMR
ppm

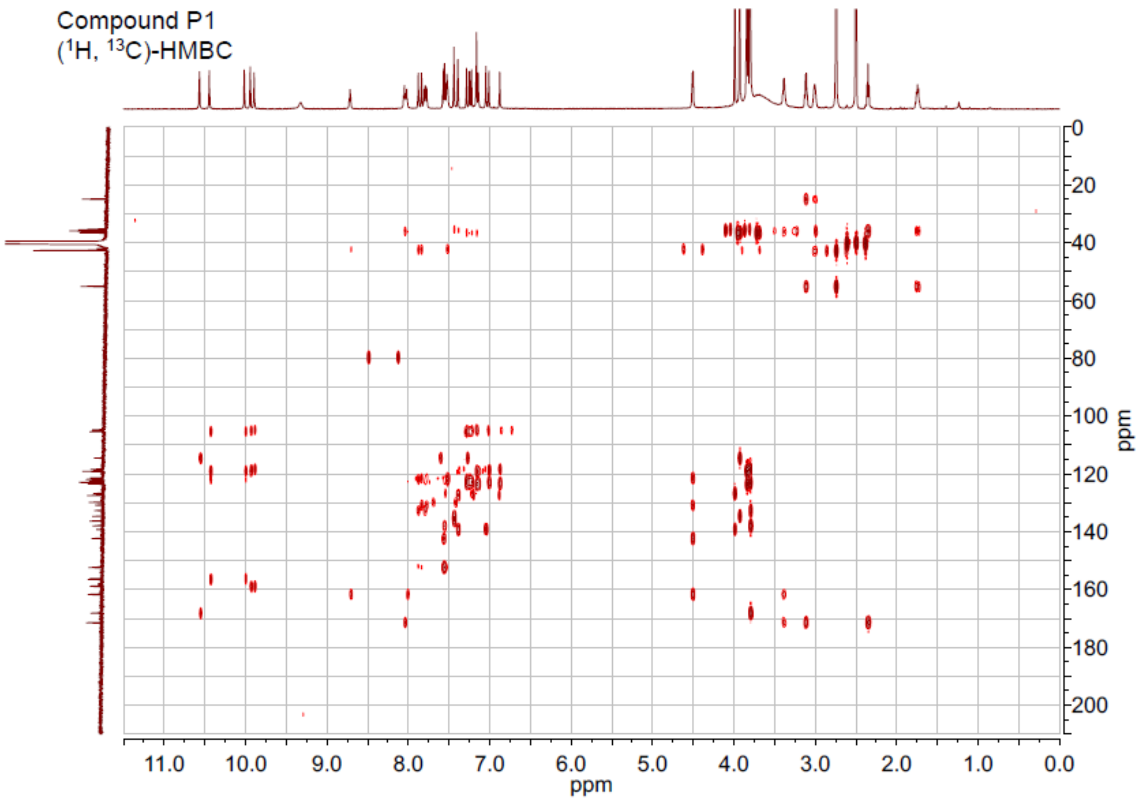
Compound P1
($^1$H, $^{13}$C)-HMBC
ppm
ppm

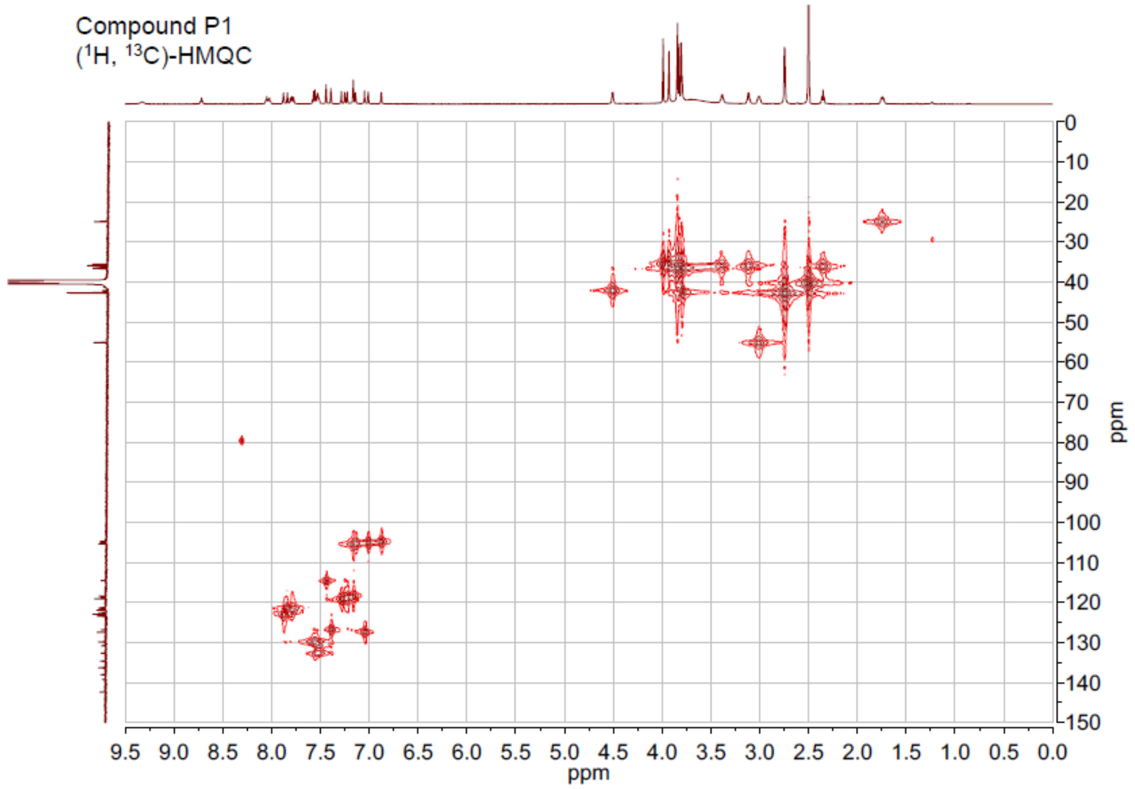
Compound P1
($^1$H, $^{13}$C)-HMQC
ppm
ppm

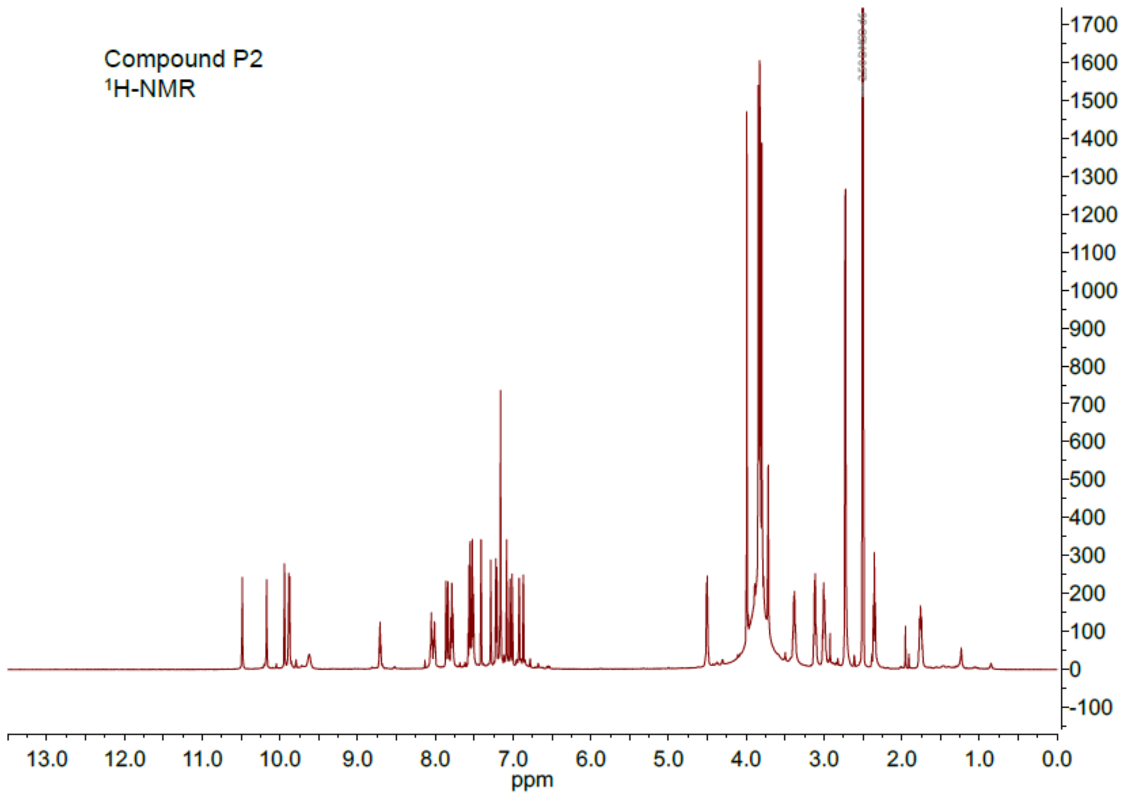
Compound P2
1H-NMR
ppm

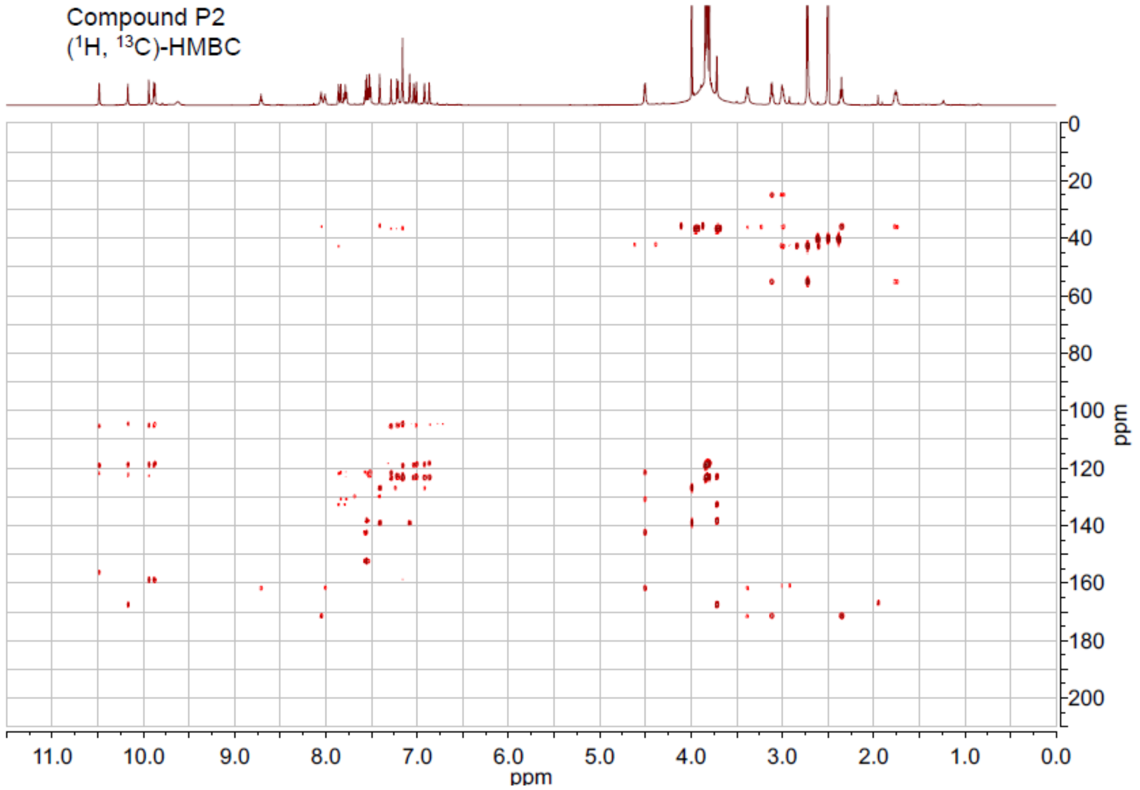
Compound P2
(1H, 13C)-HMBC
ppm

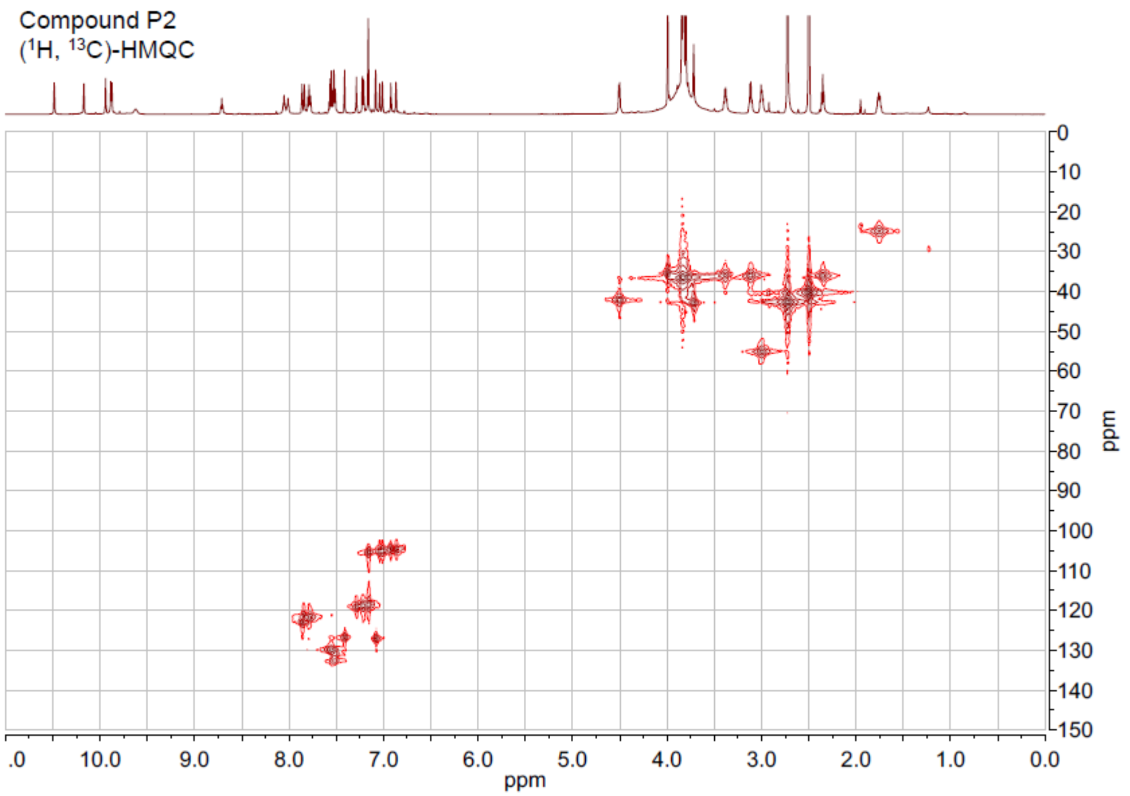
Compound P2
(1H, 13C)-HMQC
ppm
ppm

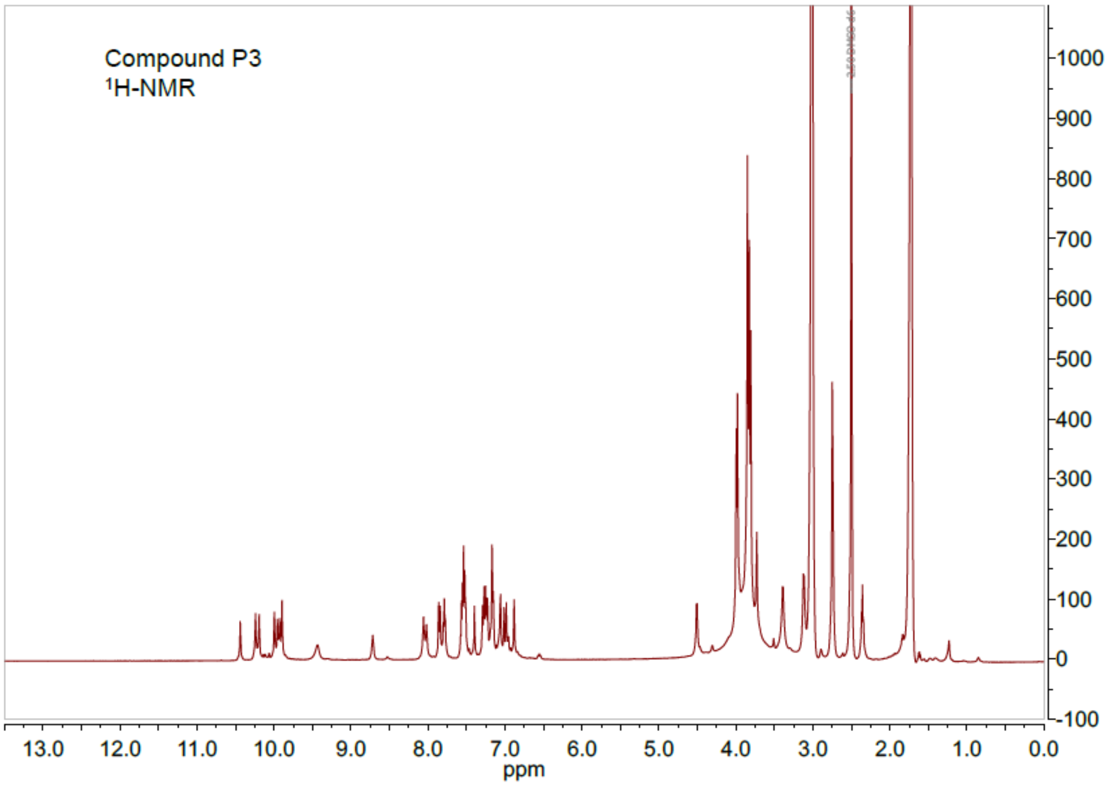
Compound P3
1H-NMR
ppm

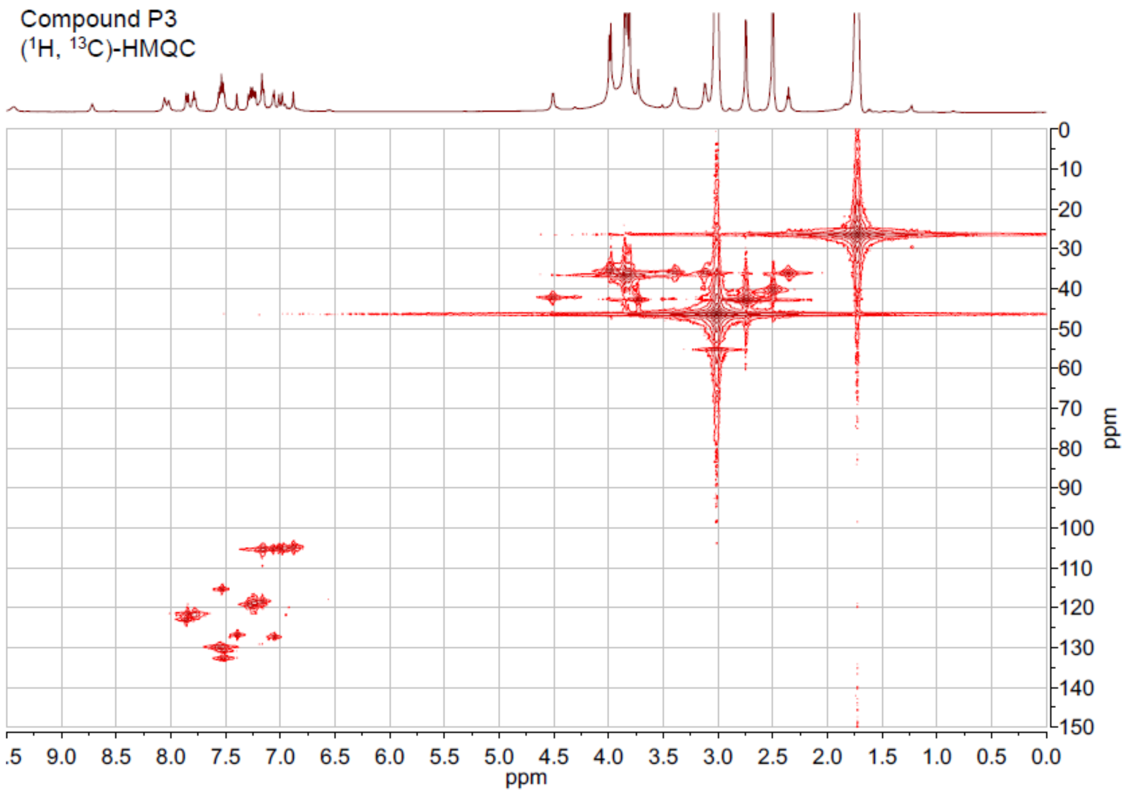
Compound P3
(1H, 13C)-HMQC
ppm

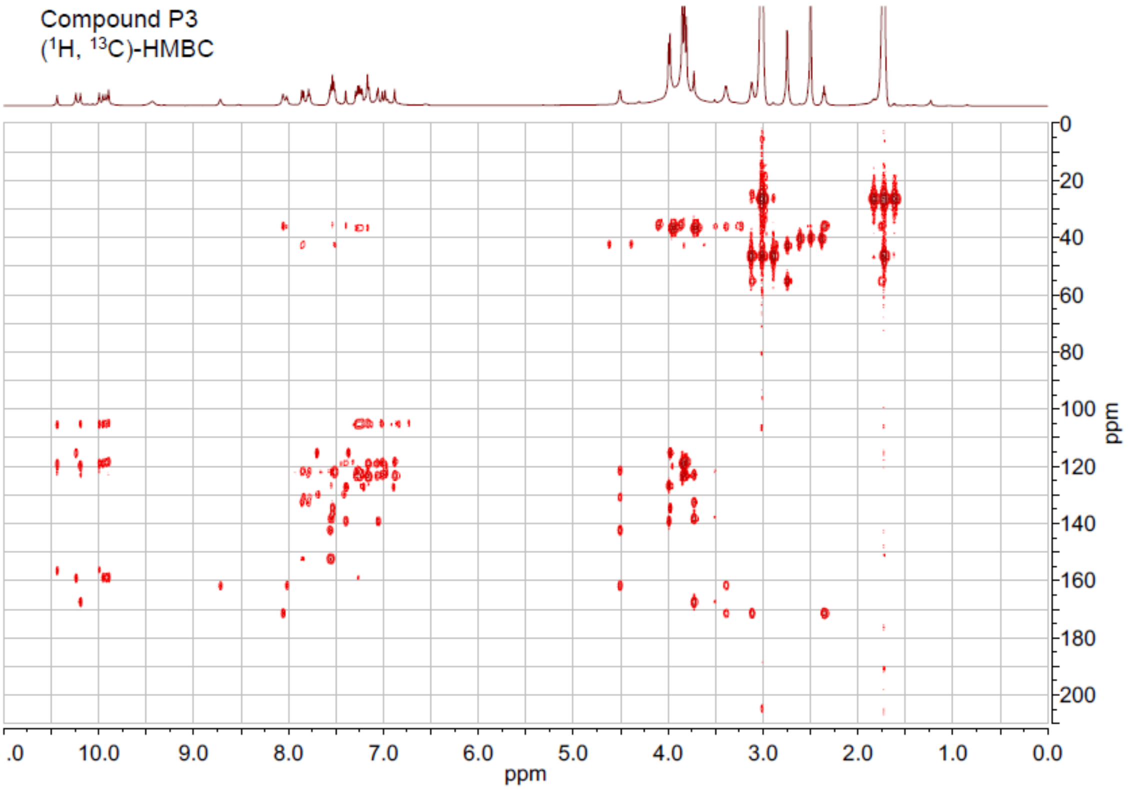
Compound P3
(1H, 13C)-HMBC
ppm

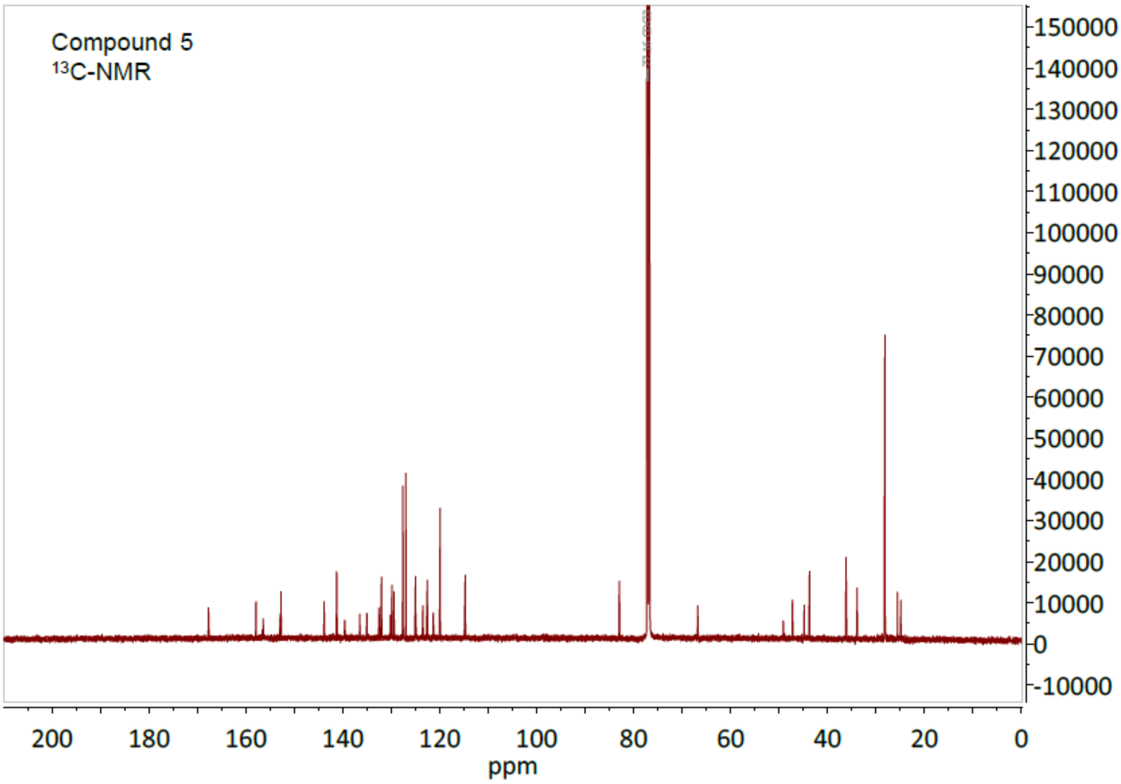
Compound 5
13C-NMR
ppm

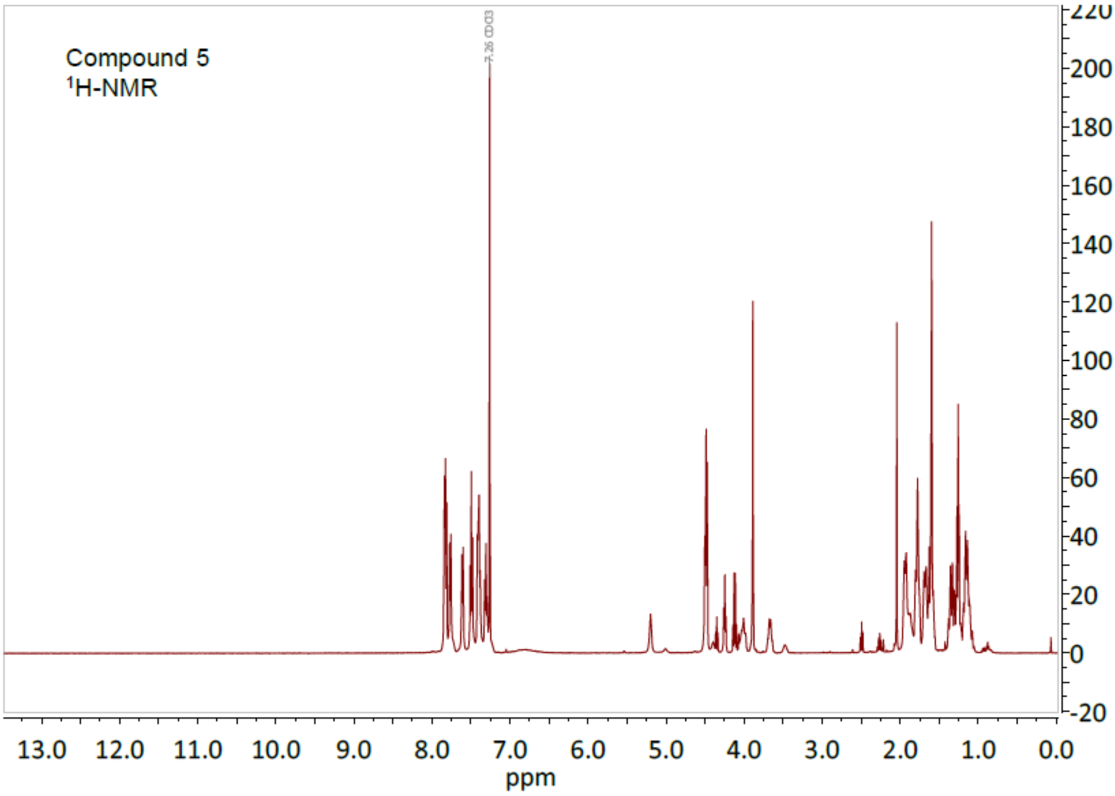
Compound 5
1H-NMR
7.26 CDCl3
ppm

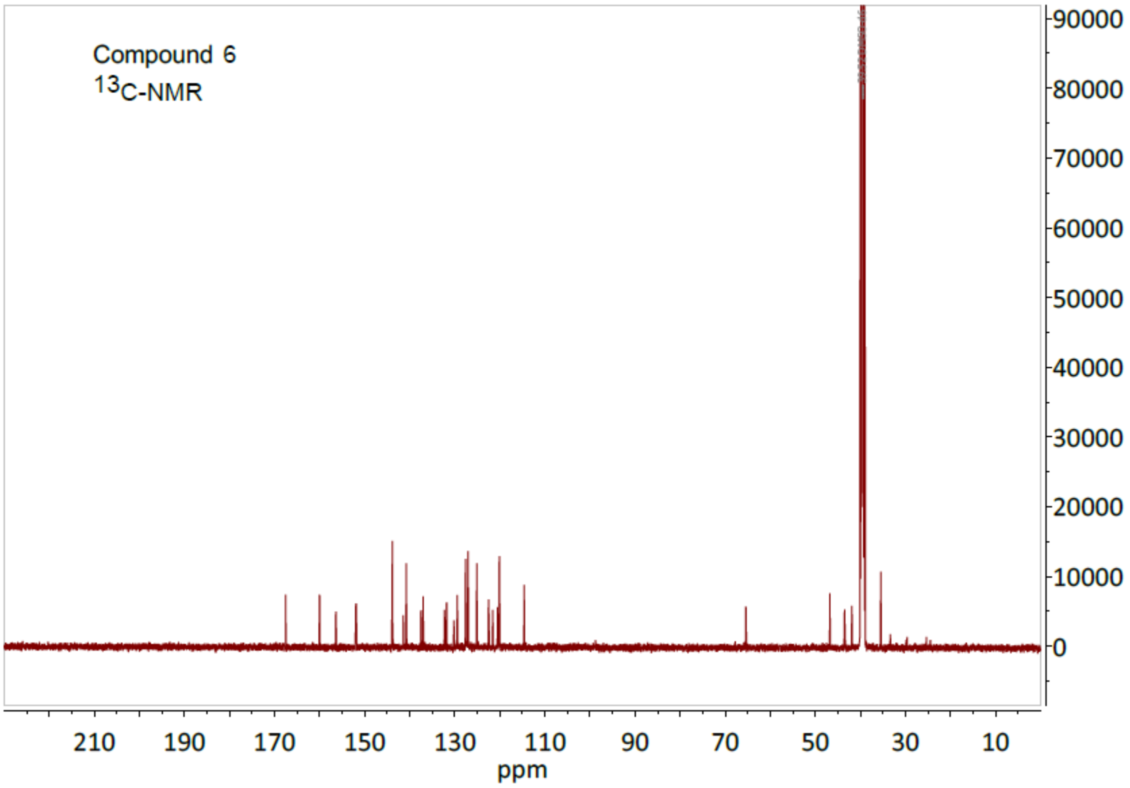
Compound 6
13C-NMR
90000
80000
70000
60000
50000
40000
30000
20000
10000
0
210
190
170
150
130
110
90
70
50
30
10
ppm

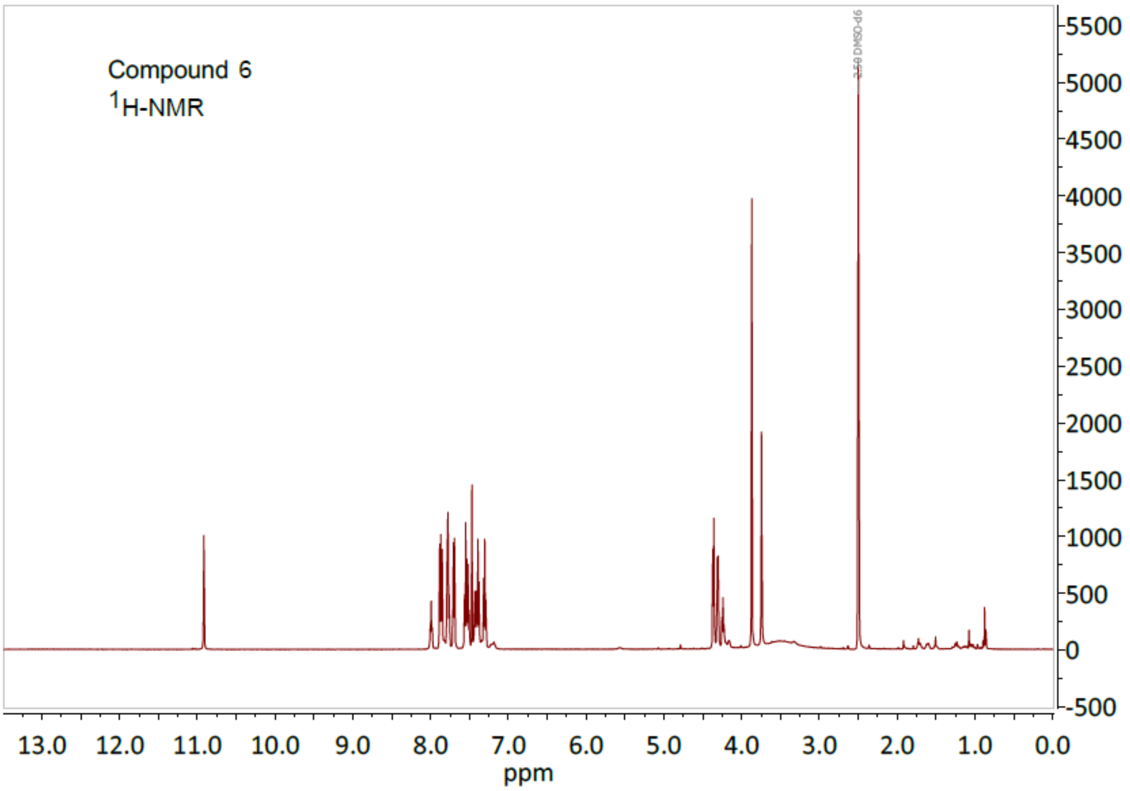
Compound 6
1H-NMR
2.50 DMSO-d6
5500
5000
4500
4000
3500
3000
2500
2000
1500
1000
500
0
-500
13.0
12.0
11.0
10.0
9.0
8.0
7.0
6.0
5.0
4.0
3.0
2.0
1.0
0.0
ppm
