# Supplementary material for: Photocontrolled DNA minor groove interactions of imidazole/pyrrole polyamides
Source: Beilstein J Org Chem. 2020 Jan 9;16:60–70. doi: 10.3762/bjoc.16.8 (PMC6964667; doi:10.3762/bjoc.16.8)
Supplement: File 3 — Photoisomerization of P1–P3 and TO displacement experiments. [file Beilstein_J_Org_Chem-16-60-s003.pdf]

BEILSTEIN JOURNAL OF ORGANIC CHEMISTRY

Supporting Information

for

Photocontrolled DNA minor groove interactions of imidazole/ pyrrole polyamides

Sabrina Müller, Jannik Paulus, Jochen Mattay, Heiko Ihmels, Veronica I. Dodero and Norbert Sewald

Beilstein J. Org. Chem. **2020,** *16,* 60–70. doi:10.3762/bjoc.16.8

Photoisomerization of P1–P3 and TO displacement experiments

License and Terms: This is a supporting information file under the terms of the Creative Commons Attribution License (https://creativecommons.org/licenses/by/4.0). Please note that the reuse, redistribution and reproduction in particular requires that the authors and source are credited.
The license is subject to the *Beilstein Journal of Organic Chemistry* terms and conditions: (https://www.beilstein-journals.org/bjoc)

^{1}H-NMR of Compound **P1**

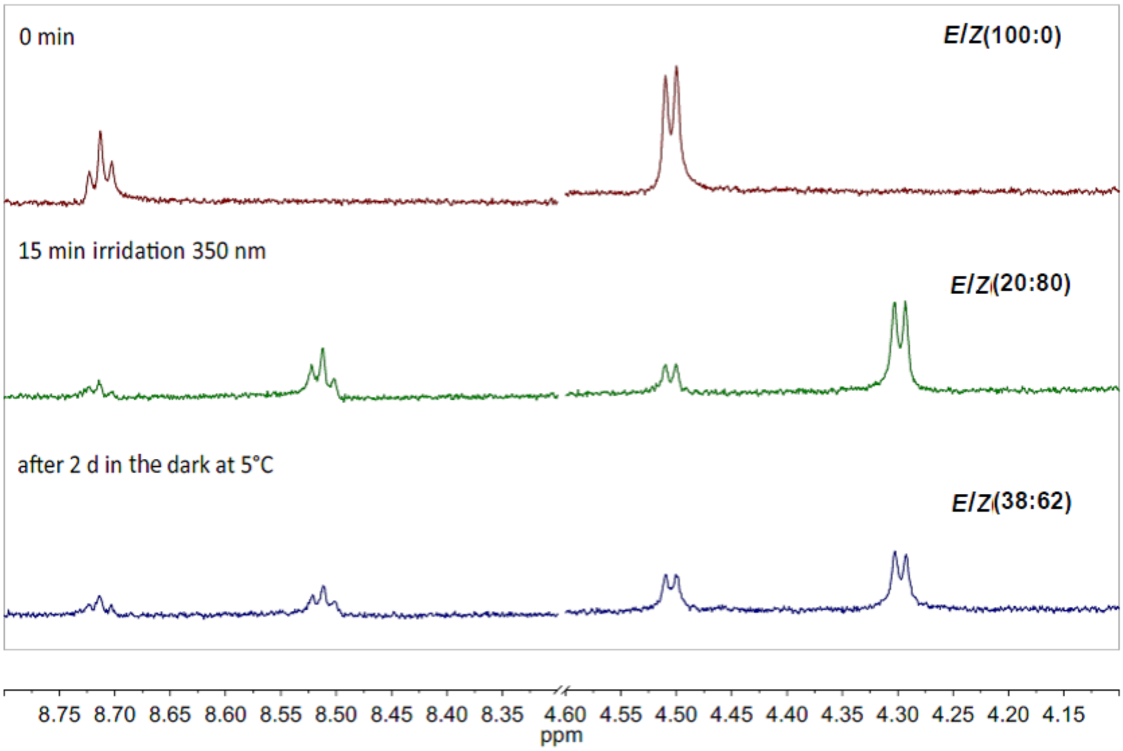

^{1}H-NMR of Compound **P2**

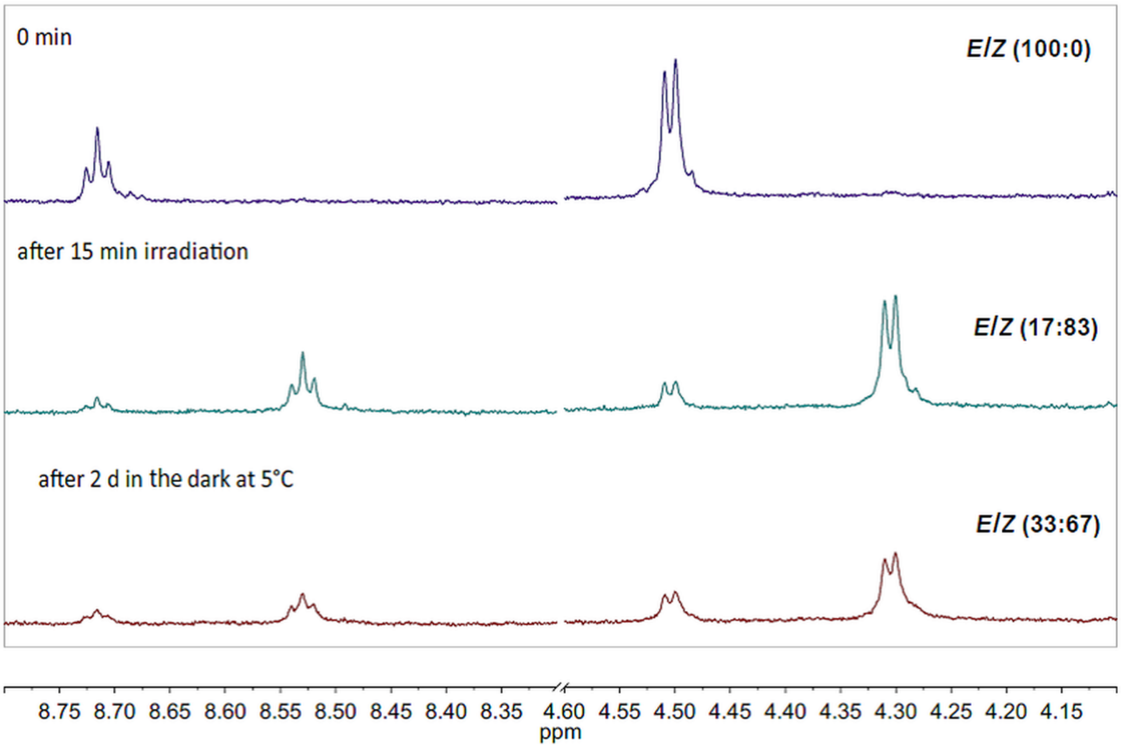

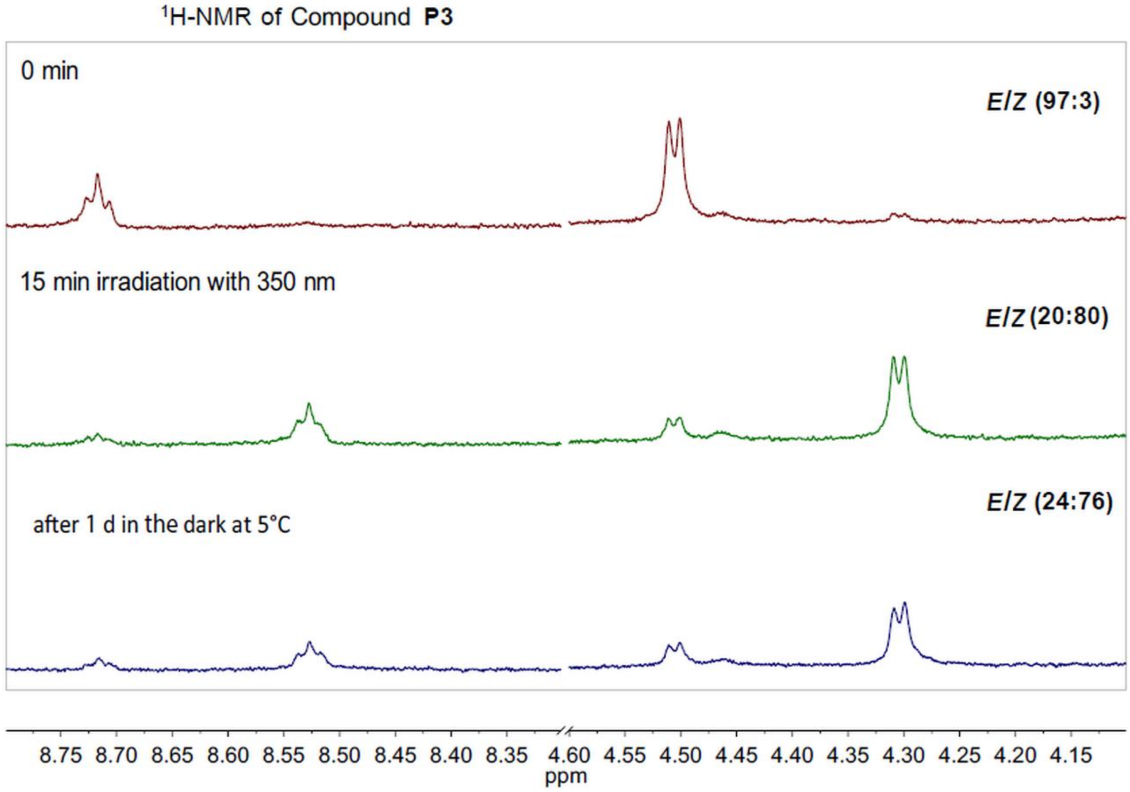

1H-NMR of Compound P3
0 min
E/Z (97:3)
15 min irradiation with 350 nm
E/Z (20:80)
E/Z (24:76)
after 1 d in the dark at 5°C
8.75 8.70 8.65 8.60 8.55 8.50 8.45 8.40 8.35 4.60 4.55 4.50 4.45 4.40 4.35 4.30 4.25 4.20 4.15
ppm

Fluorescent indicator displacement (FID) assay

A stock solution of duplex DNA was prepared from ct DNA (12.5 µL, c = 20 µmol/L in BPE buffer) and incubated (30 min) with TO (10 µL, c = 50 µmol/L). The emission spectrum was recorded from 506 nm to 750 nm (λ_{ex} = 501 nm). A solution of *E*-**P1** was added stepwise, and 3 min after each addition an emission spectrum was recorded. The fluorescence band (510-750 nm) was analyzed by integration of each emission signal (*FA*). The percentage of displacement *PD* was determined according to Eq 1.

$$\mathrm{PD} = 100 - \frac{FA}{FA_0} \cdot 100 \qquad \text{(Eq. 1)}$$

FA_0 is the integral of the emission signal before the addition of the ligand solution. PD was plotted as a function of the ligand concentration.

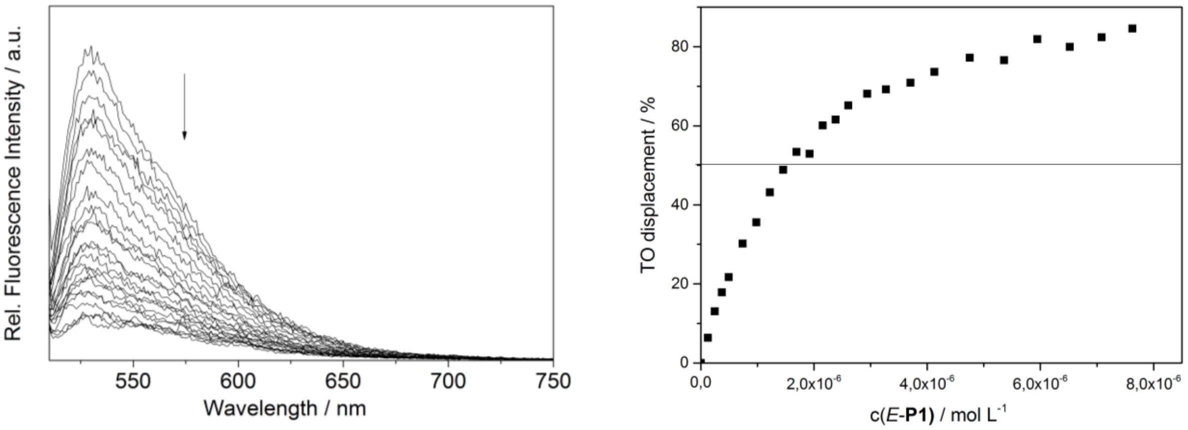

Figure S1. Left: Fluorimetric detection of the displacement of Thiazole Orange (0.5 µM) from ct DNA by addition of *E*-**P1**. Right: Plot of the percentage of displacement according to eq. 1 versus c(*E*-**P1**).
