# Supplementary material for: Photocontrolled DNA minor groove interactions of imidazole/pyrrole polyamides
Source: Beilstein J Org Chem. 2020 Jan 9;16:60–70. doi: 10.3762/bjoc.16.8 (PMC6964667; doi:10.3762/bjoc.16.8)
Supplement: File 4 — HRMS spectra of P1–P3. [file Beilstein_J_Org_Chem-16-60-s004.pdf]

BEILSTEIN JOURNAL OF ORGANIC CHEMISTRY

Supporting Information

for

Photocontrolled DNA minor groove interactions of imidazole/ pyrrole polyamides

Sabrina Müller, Jannik Paulus, Jochen Mattay, Heiko Ihmels, Veronica I. Dodero and Norbert Sewald

Beilstein J. Org. Chem. **2020,** *16,* 60–70. doi:10.3762/bjoc.16.8

HRMS spectra of P1–P3

License and Terms: This is a supporting information file under the terms of the Creative Commons Attribution License (https://creativecommons.org/licenses/by/4.0). Please note that the reuse, redistribution and reproduction in particular requires that the authors and source are credited.
The license is subject to the *Beilstein Journal of Organic Chemistry* terms and conditions: (https://www.beilstein-journals.org/bjoc)

Compound **P1**

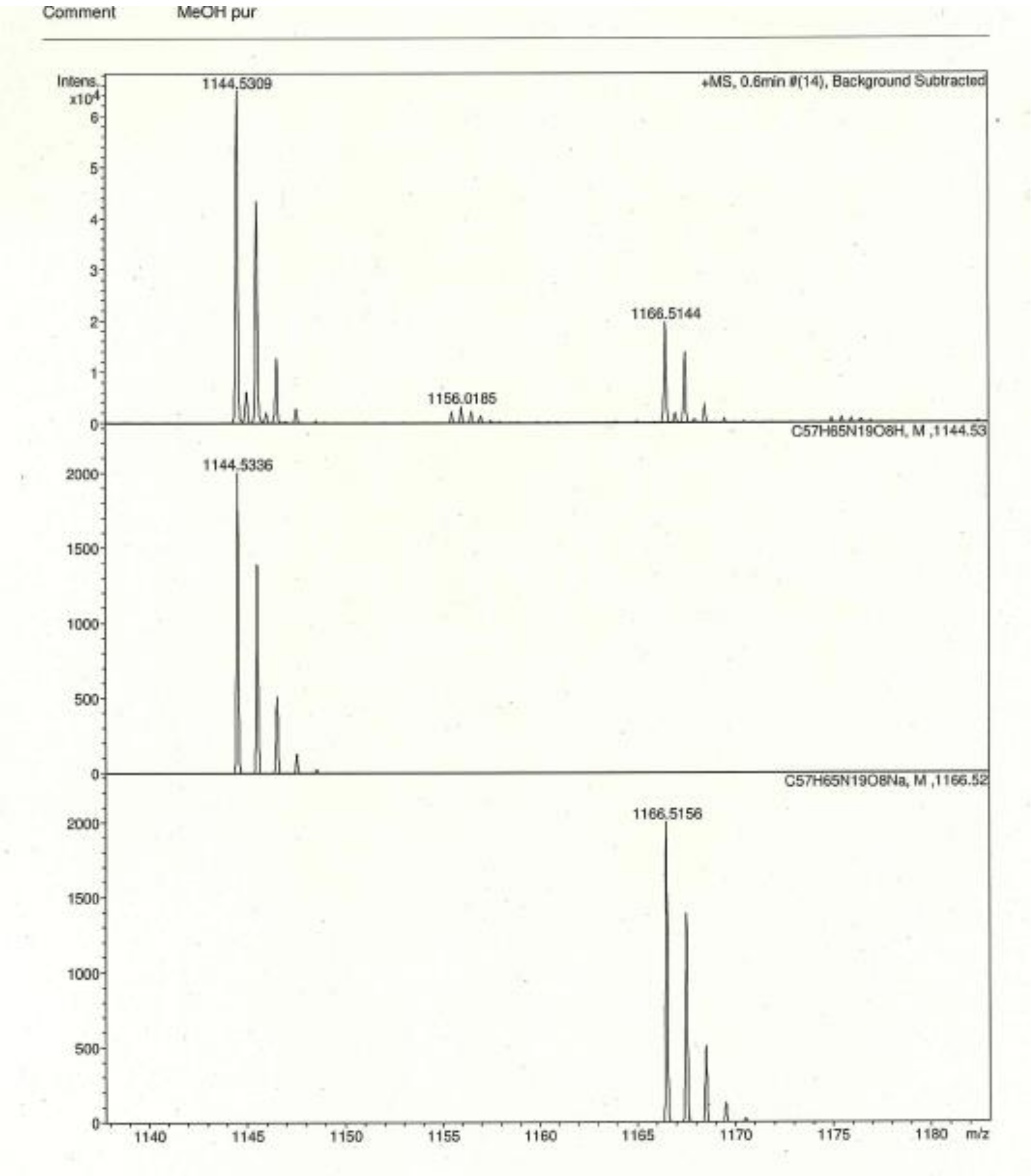

Comment MeOH pur
Intens. x10^4
1144.5309
+MS, 0.6min #(14), Background Subtracted
1156.0185
1166.5144
C57H65N19O8H, M ,1144.53
1144.5336
C57H65N19O8Na, M ,1166.52
1166.5156
m/z

Compound **P2**

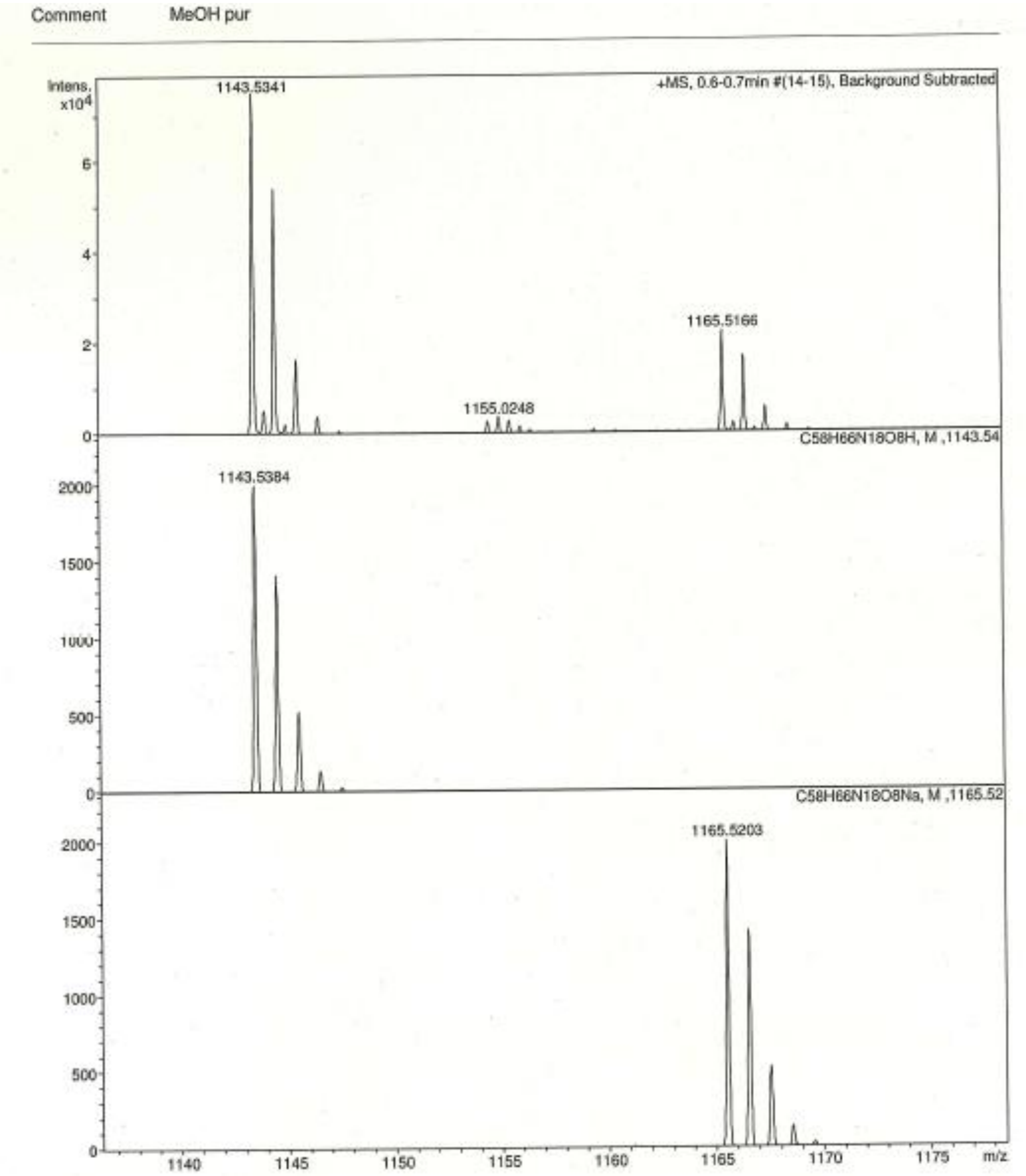
Comment
MeOH pur
Intens.
x10^4
+MS, 0.6-0.7min #(14-15), Background Subtracted
1143.5341
1155.0248
1165.5166
C58H66N18O8H, M ,1143.54
1143.5384
C58H66N18O8Na, M ,1165.52
1165.5203
1140
1145
1150
1155
1160
1165
1170
1175
m/z

Compound **P3**

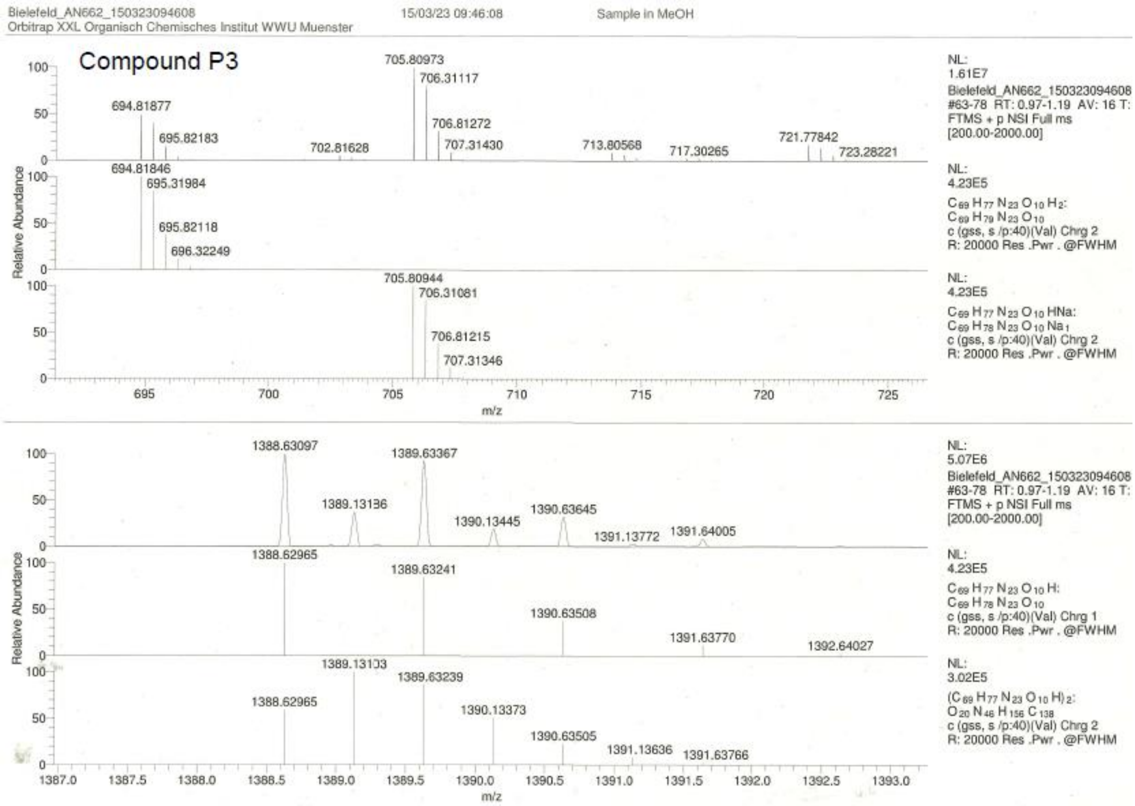

Bielefeld_AN662_150323094608
Orbitrap XXL Organisch Chemisches Institut WWU Muenster
15/03/23 09:46:08
Sample in MeOH
Compound P3
705.80973
706.31117
694.81877
706.81272
695.82183
702.81628
707.31430
713.80568
717.30265
721.77842
723.28221
694.81846
695.31984
695.82118
696.32249
705.80944
706.31081
706.81215
707.31346
Relative Abundance
m/z
NL: 1.61E7
Bielefeld_AN662_150323094608 #63-78 RT: 0.97-1.19 AV: 16 T: FTMS + p NSI Full ms [200.00-2000.00]
NL: 4.23E5
C69 H77 N23 O10 H2: C69 H79 N23 O10
c (gss, s /p:40)(Val) Chrg 2
R: 20000 Res .Pwr . @FWHM
NL: 4.23E5
C69 H77 N23 O10 HNa: C69 H78 N23 O10 Na1
c (gss, s /p:40)(Val) Chrg 2
R: 20000 Res .Pwr . @FWHM
1388.63097
1389.63367
1389.13136
1390.13445
1390.63645
1391.13772
1391.64005
1388.62965
1389.63241
1390.63508
1391.63770
1392.64027
1389.13103
1389.63239
1388.62965
1390.13373
1390.63505
1391.13636
1391.63766
NL: 5.07E6
Bielefeld_AN662_150323094608 #63-78 RT: 0.97-1.19 AV: 16 T: FTMS + p NSI Full ms [200.00-2000.00]
NL: 4.23E5
C69 H77 N23 O10 H: C69 H78 N23 O10
c (gss, s /p:40)(Val) Chrg 1
R: 20000 Res .Pwr . @FWHM
NL: 3.02E5
(C69 H77 N23 O10 H)2: O20 N46 H156 C138
c (gss, s /p:40)(Val) Chrg 2
R: 20000 Res .Pwr . @FWHM
